# Global reorganization of deep-sea circulation and carbon storage after the last ice age

**DOI:** 10.1126/sciadv.abq5434

**Published:** 2022-11-16

**Authors:** Patrick A. Rafter, William R. Gray, Sophia K.V. Hines, Andrea Burke, Kassandra M. Costa, Julia Gottschalk, Mathis P. Hain, James W.B. Rae, John R. Southon, Maureen H. Walczak, Jimin Yu, Jess F. Adkins, Timothy DeVries

**Affiliations:** ^1^ University of California, Irvine, Irvine, CA, USA.; ^2^ Laboratoire des Science du Climat et de l’Environnement (LSCE/IPSL), Université-Paris-Saclay, Gif-sur-Yvette, France.; ^3^ Woods Hole Oceanographic Institution, Woods Hole, MA, USA.; ^4^ University of St. Andrews, St. Andrews, Scotland, UK.; ^5^ Institute of Geosciences, Kiel University, Kiel, Germany.; ^6^ University of California, Santa Cruz, Santa Cruz, CA, USA.; ^7^ Oregon State University, Corvallis, OR, USA.; ^8^ Pilot National Laboratory for Marine Science and Technology (Qingdao), Qingdao 266237, China.; ^9^ Australia National University, Canberra, Australia.; ^10^ California Institute of Technology, Pasadena, CA, USA.; ^11^ Department of Geography and Earth Research Institute, University of California, Santa Barbara, CA, USA.

## Abstract

Using new and published marine fossil radiocarbon (^14^C/C) measurements, a tracer uniquely sensitive to circulation and air-sea gas exchange, we establish several benchmarks for Atlantic, Southern, and Pacific deep-sea circulation and ventilation since the last ice age. We find the most ^14^C-depleted water in glacial Pacific bottom depths, rather than the mid-depths as they are today, which is best explained by a slowdown in glacial deep-sea overturning in addition to a “flipped” glacial Pacific overturning configuration. These observations cannot be produced by changes in air-sea gas exchange alone, and they underscore the major role for changes in the overturning circulation for glacial deep-sea carbon storage in the vast Pacific abyss and the concomitant drawdown of atmospheric CO_2_.

## Teaser

A compilation of radiocarbon measurements is used to characterize deep-sea overturning since the last ice age.

## INTRODUCTION

**Fig. 1.**
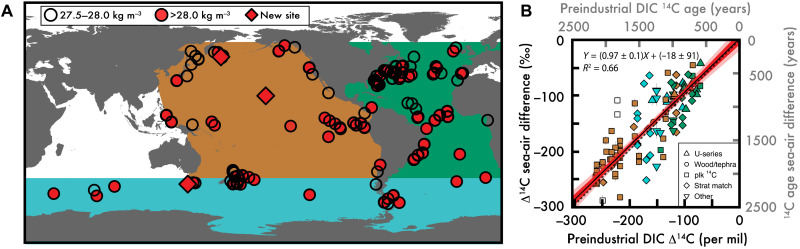
An ensemble of marine fossil ^14^C/C and a first-order test of fidelity. (**A**) Locations for all deep-sea proxy seawater ^14^C/C measurements used in this study, all of which are below the neutral density surface (γ^n^) = 27.5 kg m^−3^ (below the depth of intermediate water masses). Open symbols are mid-depth sites above or on the neutral density surface γ^n^ = 28.0 kg m^−3^. Closed symbols are γ^n^ > 28.0 kg m^−3^ “bottom water” sites. Diamonds are sites with new data provided in this study. The basin colors in (A) identify the location of measurements in (**B**), which compares preindustrial seawater dissolved inorganic carbon (DIC) from (*30*) and all compiled proxy ^14^C/C (differenced from the contemporaneous atmosphere) over the past 4 ka (see fig. S1 for additional age ranges). The dashed line in (B) is 1:1, the solid line is the slope, and the red envelope is 68 and 95% error range.

The ocean’s ability to store and release carbon via changes in biology, chemistry, and physics (*1*) makes it a prime candidate for driving changes in glacial-interglacial atmospheric carbon dioxide (CO_2_) and the global ice ages of the late Pleistocene. Physical changes in deep-sea ventilation—the combined influence of air-sea gas exchange and circulation-driven transfer of gases, including CO_2_—are especially important because they can alter the carbon storage capacity of the ocean. Several studies indicate reduced deep-sea ventilation during glacial periods (*2*–*6*), which has been linked to a slower ocean overturning rate (*7*). However, other studies suggest that the ventilation proxy data are indistinguishable from modern circulation (*8*, *9*), instead suggesting that the reduced glacial deep-sea ventilation could solely derive from reduced air-sea gas exchange in the subpolar Southern Ocean (*10*–*12*), although these studies have typically used datasets that poorly constrain the Pacific Ocean. Differentiating between these two drivers of glacial deep-sea ventilation is valuable for determining the climate and carbon cycle dynamics underpinning glacial-interglacial variations in CO_2_. If glacial ocean circulation was no different from today, the apparent glacial reduction in deep-sea ventilation and amplification of ocean carbon sequestration would derive from relatively small-scale, high-latitude surface ocean conditions. This contrasts with reduced glacial deep-sea ventilation and deep ocean carbon storage that is compelled by basin- or even global-scale change in overturning circulation. Here, using a compilation of new and published marine fossil radiocarbon (^14^C) measurements (Fig. 1A), we reconstruct glacial-interglacial marine ^14^C/C along the density surfaces of modern ocean overturning in all major ocean basins over the past 25,000 years (25 ka). When compared to new stable carbon isotope measurements and numerical model results, our deep-sea ^14^C/C dataset suggests that glacial ventilation of the Pacific Ocean, the largest ocean basin on Earth and therefore a key carbon reservoir, was profoundly different from today and cannot be solely explained by a change in air-sea gas exchange.

The radiocarbon content of seawater (^14^C/C, commonly expressed as a ^14^C age or as Δ^14^C; see the Supplementary Materials for more information) has long been recognized as a “most useful tracer” (*13*) of ocean circulation and ventilation. This is because ^14^C is introduced to the global ocean via the air-sea exchange of CO_2_, and as surface waters descend into the ocean interior, seawater ^14^C/C is progressively lowered only by radioactive decay or mixing with water masses with lower ^14^C/C levels. Therefore, outside of rare instances of significant geologic carbon addition from the seafloor (*14*, *15*), the ^14^C/C of seawater reflects the same processes affecting the partitioning of CO_2_ between the ocean and the atmosphere, i.e., changes in ocean biology, chemistry, and physics [see (*1*)]. There are various methods for interpreting marine fossil ^14^C/C [see (*16*–*19*)], and here, we use the conventional “marine fossil–minus–the contemporaneous atmosphere” method because it is the simplest approach that is applicable to all observations, including both benthic foraminifera and deep-sea corals. As per convention, this “^14^C ventilation age” is used throughout our study. All new and published planktic foraminifera ^14^C age models (those requiring calibration to atmospheric ^14^C/C) were updated to the most recent IntCal20 atmospheric ^14^C/C (*20*), with regionally appropriate reservoir ages (see the Supplementary Materials). This multisubstrate archive allows us to provide a comprehensive view of ^14^C/C in the deep Atlantic, Southern, and Pacific Oceans. A paucity of glacial-interglacial ^14^C/C observations from the deep Indian Ocean (Fig. 1A) does not allow us to estimate basin-scale values, and this region will not be further discussed in our study.

## MATERIALS AND METHODS

New radiocarbon analyses were made using sediment cores from the subarctic North Pacific, central North Pacific, and Subantarctic sites (see diamonds in Fig. 1A and fig. S4). Each sample was washed using deionized water in a 63-μm sieve, and mixed benthic foraminifera species (notably without *Pyrgo* spp.) were selected from the >250-μm fraction. Samples were graphitized using standard Keck Carbon Cycle Accelerator Mass Spectrometry Laboratory methods at University of California, Irvine, including a 10% leach of each sample to remove potential secondary calcite. More details on the pretreatment, graphitization, and assigning the calendar ages for sample depths are detailed in the Supplementary Materials.

## RESULTS

On the basis of this ensemble of measurements (Fig. 1A), we find that marine fossil ^14^C/C recorded since 4 ka before the present (BP) [as in (*21*)] from all depths compares well with pre-1950 seawater (pretreated by removing ^14^C influenced by 20th-century thermonuclear weapons testing; see the Supplementary Materials) (Fig. 1B). The seawater-proxy ^14^C/C comparison in Fig. 1B illustrates the classic global ocean “conveyor belt” (*22*), where the Atlantic (green) has the highest and the Pacific (brown) has the lowest seawater ^14^C/C (low Δ^14^C or old ^14^C ventilation age), providing a first-order validation of the ^14^C ventilation proxy (Fig. 1A). Additional sensitivity analyses (fig. S1) indicate a robust, linear relationship between marine fossil ^14^C/C and preindustrial seawater ^14^C/C, with slopes varying from 0.96 ± 0.1 to 1.03 ± 0.1 and *R*^2^ value from 0.60 to 0.82 (all with *P* << 0.001). The age models used by each study are shown as symbols in Fig. 1B (and fig. S1) and indicate that age model assumptions do not impose a systematic bias on our results.

### Modern deep-sea ^14^C ventilation ages and global overturning circulation

**Fig. 2.**
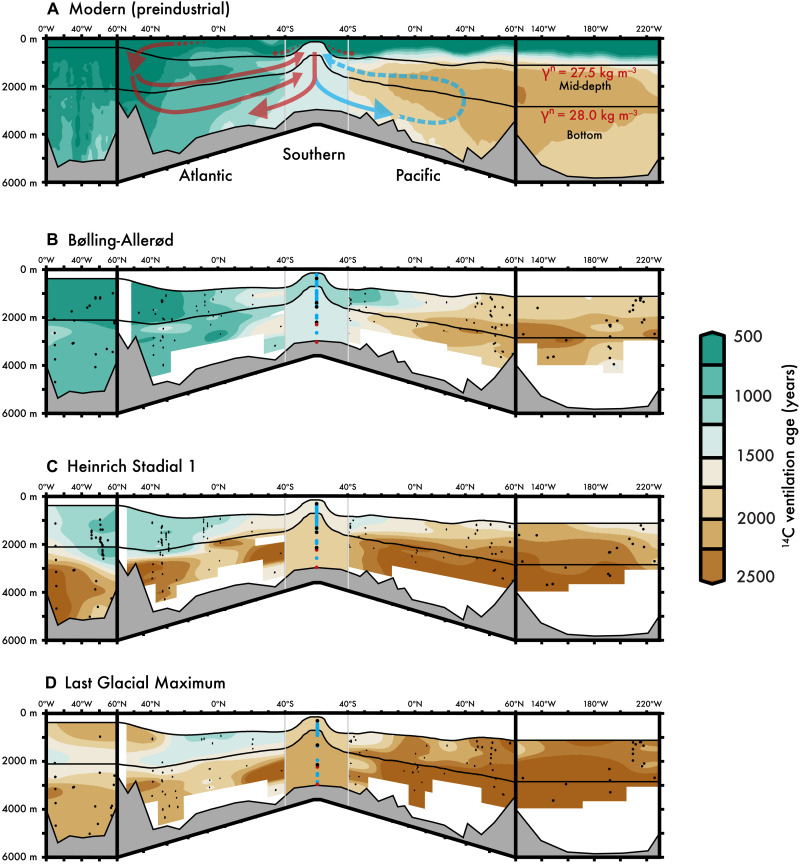
Deep-sea ^14^C ventilation ages of the LGM and early deglaciation were much older than today. This plot shows the ^14^C ventilation age of Atlantic, Southern, and Pacific Ocean seawater and is composed of five panels, from left to right: meridional-average North Atlantic (20°N to 60°N), zonal-average Atlantic (60°N to 40°S), Southern Ocean average within chosen density surfaces, zonal-average Pacific (60°N to 40°S), and meridional-average North Pacific (20°N to 60°N). Note that the size of each ocean basin’s end piece—the tips of the boomerang—and the width of the Southern Ocean are scaled to the volume of each ocean (the Pacific being >2 times the volume of the Atlantic). Symbols are site depths (color-coded for Southern Ocean density averages: Atlantic, red; Indian, black; Pacific, blue). The modern ocean ^14^C ventilation age (**A**) [data from (*15*, *57*)] is considerably younger than the (**D**) LGM (23 to 18 ka BP) and (**C**) the first stage of deglacial global warming (HS1; 18 to 14.7 ka BP). However, by the (**B**) BA (14.7 to 12.8 ka BP), global ^14^C ventilation ages approach modern values. Intermediate-depth ^14^C ventilation ages are not shown because these are not directly reflecting the overturning of the upper and lower cells and have known influences from the input of geologic carbon (*21*). The black lines represent neutral density (γ^n^) surfaces of 27.5 and 28 kg m^−3^ and provide a rough approximation for the upper and lower cells of the modern figure 8 circulation [arrows in (A)].

The relatively young Atlantic and old Pacific ^14^C ventilation ages in Fig. 1B arise from the overturning of upper and lower circulation cells that intertwine like the loops of a “figure 8” (*23*). We show this circulation and modern Atlantic, Southern, and Pacific ^14^C ventilation ages as a “boomerang” plot in Fig. 2A to emphasize both the Southern Ocean’s central role within the interlocking loops of the figure 8 and the vastly different ocean volumes (the Southern Ocean is simplified to average values between density surfaces in all plots of Fig. 2). Following the arrows in Fig. 2A, the formation of the North Atlantic Deep Water (NADW) introduces well-equilibrated surface waters (and therefore young ^14^C ventilation ages) to the deep sea [here defined as greater than or equal to the 27.5 kg m^−3^ neutral density surface (γ^n^)]. These well-equilibrated Atlantic waters move south, joining older ^14^C age Indian and Pacific Deep Water in the deep Southern Ocean (*23*). These mixed Southern Ocean waters (*24*) upwell along isopycnals to the Southern Ocean surface, and the least dense waters (here, γ^n^ < 27.5 kg m^−3^) move equatorward (*25*), eventually reconnecting with the North Atlantic surface (see dots in Fig. 2A). The densest of these upwelled Southern Ocean waters spend a relatively brief time at the surface such that the ^14^C ventilation age is not reset to the contemporaneous atmosphere (*26*, *27*), making this relatively small subpolar region an important zone for deep-sea ventilation (*10*–*12*). The densification of these subpolar surface waters on the periphery of the Antarctic continent forms Antarctic Bottom Water (AABW) (*28*, *29*), here defined as γ^n^ > 28 kg m^−3^ (see the Supplementary Materials). Because they have not fully equilibrated with the atmosphere, AABW introduces a large “preformed” ^14^C ventilation age to global bottom waters (*26*). The closure of this modern deep-sea overturning (i.e., the reconnection of the upper and lower loops of the figure 8) occurs between the Pacific bottom and mid-depth waters via diapycnal mixing (*23*) (dashed blue arrow in Fig. 2A). Since there is no deep-water formation in the modern North Pacific, this diapycnal mixing is one of the only mechanisms for delivering ^14^C/C to the mid-depth Pacific, placing it downstream of the abyssal Pacific. The result of this modern ocean overturning circulation is that the modern mid-depth Pacific Ocean—not Pacific bottom water—has the oldest ^14^C ventilation ages of any water mass in the modern ocean.

## DISCUSSION

### Reduced deep-sea ventilation during the LGM

Our compilation of new and published marine fossil ^14^C/C measurements illustrate that the ^14^C ventilation age of the deep Atlantic, Southern, and Pacific Oceans were collectively much older during the Last Glacial Maximum (LGM; 23 to 18 ka BP) than today (Fig. 2D and fig. S2). Following the LGM, our data show a general decrease in ^14^C ventilation age for most deep-sea basins in time slices during the deglaciation (Fig. 2, B and C). To quantify the basin-scale changes in ^14^C ventilation, we apply a simple depth-bias correction as well as locally weighted/estimated scatter plot smooting (LOESS) smoothing of our proxy data over the past 25 ka (tables S1 and S2; details in the Supplementary Materials). LOESS provides a conservative estimate of the deep-sea ^14^C ventilation age because it applies different weights to values according to their distance from the binned mean value while also considering the overall trend of the time series when calculating the best fit. We propagate calendar age and analytical uncertainties through to our final LOESS uncertainties (see the Supplementary Materials for more detail and for the error propagation method). The data density is high enough that we also parse the ^14^C/C using the characteristic γ^n^ ranges of the upper and lower loops of the modern figure 8 configuration of ocean overturning: “mid-depth” waters having γ^n^ = 27.5 to 28 kg m^−3^ and “bottom” waters with γ^n^ > 28 kg m^−3^ (Fig. 2). (Note that our bottom water proxy observations are generally limited to the <4000-m-depth range for the Pacific and therefore primarily capture the shallower depths of these waters.)

**Fig. 3.**
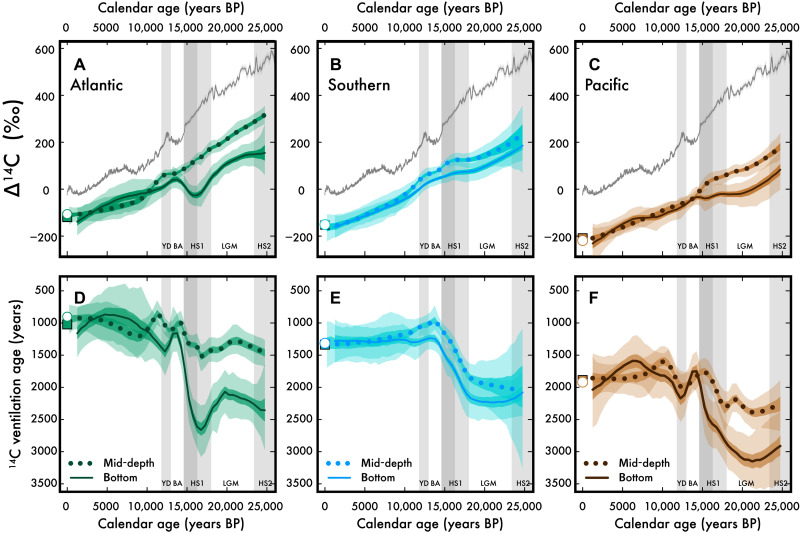
Last glacial and deglacial ^14^C ventilation age in the deep Atlantic, Southern, and Pacific Oceans, respectively. (**A** to **C**) Decay-corrected ^14^C/C (Δ^14^C) for the atmosphere (gray line) is shown alongside mid-depth (γ^n^ = 27.5 to 28 kg m^−3^; dotted colors) and bottom water (γ^n^ > 28 kg m^−3^; solid colors) LOESS Δ^14^C trends (Materials and Methods) for the (A) Atlantic, (B) Southern, and (C) Pacific Oceans. (**D**) to (**F**) show the same data but differenced from the contemporaneous atmosphere (a.k.a., the ^14^C ventilation age). The bottom water ^14^C/C trends are lower than mid-depth water ^14^C/C (dotted) in all basins before and during the LGM, a reversal of the modern relationship (symbols) in the Pacific. HS2 is Heinrich Stadial 2. Error envelopes show 68 and 95% confidence interval from bootstrapping/Monte Carlo simulation (Materials and Methods).

With these basin-scale density surface estimates, we observe that not only are LGM deep-sea ^14^C ventilation ages older (Fig. 3), but there is also a clear separation between mid-depth and bottom water values (tables S1 and S2). We estimate a volume-weighted global bottom water ^14^C ventilation age (see the Supplementary Materials) during the LGM of 2350 ± 150 years, equivalent to a ventilation age that is 1050 ± 150 years older than the preindustrial deep sea [calculated from Global Ocean Data Analysis Project (GLODAP)v2 (*30*)]. Lower glacial atmospheric CO_2_ alters the air-sea carbon isotopic equilibration, driving an additional ≈250-year ^14^C aging of the global surface ocean (*31*, *32*), but this is minor relative to the observed aging of the volume-weighted near-global bottom waters. Our LGM-versus-modern deep-sea ^14^C age estimate is significantly older than prior estimates of ≈600 years [for depths >2000 m (*6*)] and ≈690 ± 50 years [for depths >3000 m (*33*)], which may partly be explained by our improved coverage from all regions, especially the Pacific Ocean (Fig. 1A and see figs. S2 to S4, color-coded to the original compilation; also see further discussion in the Supplementary Materials). This updated view of LGM deep-sea radiocarbon age brings our understanding of ^14^C ventilation ages in line with lower deep-sea oxygenation (resulting from increased respired carbon) (*2*–*4*), which are the combined impacts of reduced ventilation and continued remineralization of organic matter produced in the surface ocean (*34*).

### A “flipped” Pacific and slower deep overturning circulation during the LGM

One prominent observation of our deep-sea ^14^C/C records is that the oldest ^14^C ventilation ages in the glacial ocean are found in Pacific bottom waters instead of Pacific mid-depth waters as we see today (see Fig. 2A or the symbols in Fig. 3). This flipped glacial Pacific ventilation is apparent in estimated LGM Pacific mid-depth and bottom ventilation ages (tables S1 and S2). We also constructed depth profiles of ^14^C ventilation age for all basins during the LGM (fig. S2), which show that both the glacial Pacific and glacial Atlantic have ^14^C ventilation age profiles that increase with depth. Our conclusion is not sensitive to time-slice boundaries, with the full 25-ka time series showing that the flipped Pacific ventilation persists from at least 25 ka BP through the early deglaciation (Fig. 3).

Because the observation of glacial Pacific deep-sea ventilation is a potentially transformative benchmark for ocean climate modeling, we applied a series of sensitivity tests to establish the robustness of these observations. These tests show that different age model assumptions, density surfaces, data pretreatment, and more (figs. S5 to S8) consistently show a flipped Pacific ^14^C ventilation pattern during the LGM. Additional tests exclude low–sedimentation rate sites (<2 cm ka^−1^; fig. S9), but the flipped Pacific pattern persists—all indicating a significant difference between LGM Pacific mid-depth and bottom water ^14^C ventilation ages (see *t* test results in the Supplementary Materials). These tests all confirm that our original finding is not an artifact of data biasing or processing and that the flipped Pacific deep-sea ^14^C ventilation is a robust feature of the glacial ocean.

New and published measurements of the stable isotopic composition of glacial North Pacific waters (δ^13^C) (fig. S2D) are also flipped (*35*–*37*), reinforcing a large-scale change in Pacific circulation. The absolute value of seawater δ^13^C reflects the combined effects of upper ocean assimilation of carbon during photosynthesis (increasing δ^13^C) and the subsurface remineralization of organic matter (lowering δ^13^C). Today, both processes work to produce a North Pacific water column profile where the lowest values (<−0.5 per mil) are found in the uppermost mid-depth density surface (top dashed line in fig. S2D), which includes the uppermost depths of the oldest ^14^C ventilation ages in the modern mid-depth Pacific (1000 to 1500 m). However, as seen for glacial Pacific ^14^C ventilation ages, the glacial Pacific δ^13^C minimum is also flipped relative to today, with the lowest values found in Pacific bottom waters (fig. S2D). Thus, both the radioactive and stable carbon isotopic composition of glacial Pacific seawater indicate that the most poorly ventilated waters were found in the deep glacial ocean.

The pattern of glacial Pacific Ocean ^14^C ventilation cannot be produced by changing the air-sea exchange of CO_2_ because this would simply increase deep-sea ^14^C ages without changing the spatial relationships inherent to the modern overturning circulation. This is broadly tested with a recent modeling study (*38*) showing that Pacific mid-depth ^14^C ventilation ages become older with decreasing Southern Ocean air-sea gas exchange, but the relative mid-depth to bottom water relationship is the same as today. The only way to develop the observed flipped Pacific ^14^C ventilation ages, based on those model results, is to alter global overturning circulation [in (*38*), via changes to Southern Ocean buoyancy forcing].

### Southern, Atlantic, and Pacific deep-sea ventilation during the LGM

**Fig. 4.**
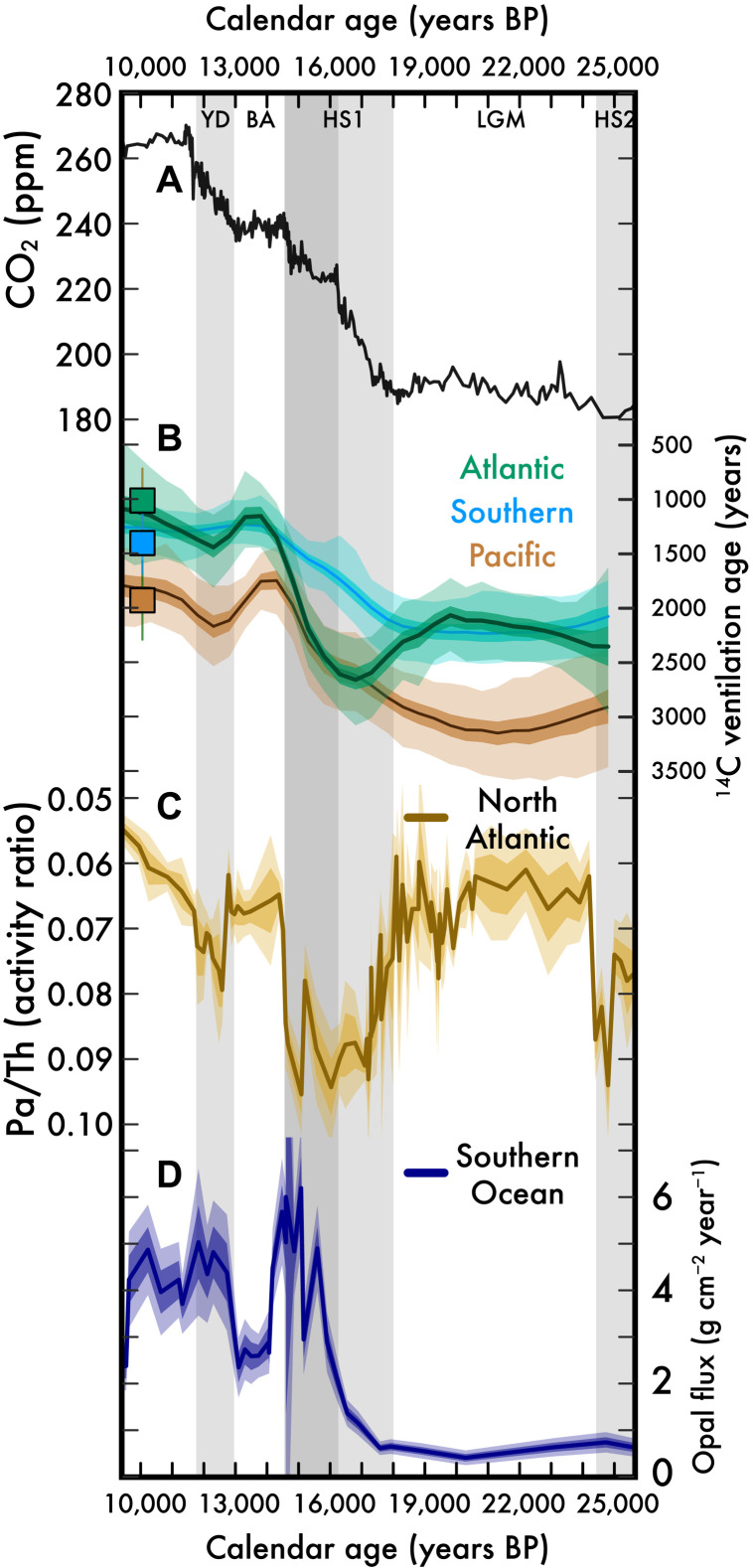
The glacial-to-deglacial relationship between atmospheric CO_2_, deep-sea ^14^C ventilation, Atlantic overturning, and Southern Ocean primary production. (**A**) Atmospheric CO_2_ from compiled Antarctic ice core records (*61*). (**B**) Reconstructed Atlantic, Southern, and Pacific Ocean bottom water ^14^C ventilation ages (this study; neutral density > 28 kg m^−3^, as in Fig. 3). Symbols are preindustrial averages (*50*). (**C**) North Atlantic overturning variation reconstructed from ^231^Pa/^230^Th records. Brown line and yellow envelope are the trend and SE from the Bermuda Rise (*52*). (**D**) Southern Ocean opal flux (*52*). ppm, parts per million.

In addition to a flipped glacial Pacific ventilation, the ^14^C ventilation ages of Southern and Pacific bottom waters—the pathway for the densest ocean waters—indicate a substantially slower glacial overturning during the LGM. It is well known that Pacific Ocean bottom water ^14^C age incorporates both preformed ^14^C aging (from reduced air-sea gas exchange in subpolar Southern surface waters) and in situ ^14^C decay during advection from the Southern Ocean (*26*). However, taking the difference between Southern and Pacific bottom water ^14^C ages should only reflect the in situ decay during advection. The preindustrial Southern-to-Pacific bottom water ^14^C age difference is 550 ± 75 years (symbols in Fig. 3), and we similarly estimate (using our LOESS estimates) a proxy-based late Holocene Southern-to-Pacific bottom water ^14^C age difference of 550 ± 150 years (over the last 4000 years BP). By contrast, the glacial Southern-to-Pacific bottom water ^14^C age difference is as high as 900 years, averaging 850 ± 150 years during 25 to 18 ka BP (Figs. 3 and 4). This estimated ≈300-year increase in transit time between Southern Ocean and Pacific bottom waters during the last glacial period is similar to the predicted lengthening of LGM “mean transit time” (*6*), both of which can be best explained by a slower rate of overturning during the LGM (*39*).

The features of glacial Pacific deep-sea ^14^C ventilation that we reconstruct are difficult to reconcile with suggestions that there was no change in glacial ocean circulation (*10*, *11*) and are more consistent with model studies linking altered glacial deep-sea overturning to increased deep-sea CO_2_ storage during the LGM (*7*). Both the glacial Atlantic and the Pacific are better characterized by a distinct two-cell overturning [as suggested in (*38*)], with older bottom water ^14^C ventilation ages overlain by better ventilated waters above ≈2000 m. This overturning structure was previously inferred for the glacial Atlantic using carbon and oxygen isotope proxy distributions (*40*, *41*), representing northern-sourced waters overlying predominantly southern-sourced waters at depth.

The fact that comparable ^14^C ventilation and δ^13^C patterns exist in both the glacial Atlantic and Pacific [also seen in oxygen isotopes (*42*)] points to a similar explanation of northern-sourced waters overlying southern-sourced waters. For example, the ^14^C ventilation age of the mid-depth Pacific during the LGM is essentially equal to today (note the similarity between 1000 and 2000 m in fig. S2C), in stark contrast with the increased ^14^C ventilation ages of all other glacial mid-depth waters (Fig. 3 and table S1). The much older (and therefore lower ^14^C/C) of glacial Pacific bottom waters cannot be the primary source of these relatively younger mid-depth Pacific ^14^C ages during the LGM. Instead, the relatively young Pacific mid-depth ^14^C ages during the LGM could instead be caused by an expansion and/or deepening of modern North Pacific Intermediate Water to depths of ≈2000 m (*35*, *42*), bringing recently ventilated waters well below the depth of the modern 27.5 kg m^−3^ density surface. This potential “North Pacific Deep Water” would drive a relative increase in the ventilation of mid-depth water, but not bottom water in the Pacific (*43*, *44*), akin to the model proposed for the LGM Atlantic (*40*). Consistent with improved Pacific Ocean mid-depth ventilation during the LGM, we see that ^14^C ventilation ages above the mid-depth North Pacific (averaged from 20°N to 60°N in Fig. 2D, right side) are reduced toward the surface during the LGM. Crucially, the suggested enhancement of North Pacific overturning here and in (*35*, *42*) also explains the much higher North Pacific mid-depth δ^13^C during the LGM (fig. S1).

These changes in glacial deep-sea ^14^C ventilation ages underscore the important contribution of changing deep-sea circulation to establishing the low glacial values of atmospheric CO_2_ (Fig. 4A), a deep-sea modification that would work to magnify related CO_2_ sequestration processes of reduced Southern Ocean air-sea gas exchange (*10*–*12*) and increased carbon fixation and export to the deep ocean (*45*).

### Deep-sea reorganization and carbon ventilation during the deglaciation

At the onset of deglaciation, around 18 ka BP, there was a synchronous increase in deep Southern Ocean ventilation (reduced ^14^C ventilation ages) and atmospheric CO_2_ rise (Fig. 4), a relationship that is consistent with the Southern Ocean as the critical regulator of ocean-atmosphere CO_2_ partitioning (*10*–*12*, *46*–*48*). However, we also find that both Pacific mid-depth and bottom water ^14^C ventilation ages are decreasing at this time. This deglacial Southern and Pacific Ocean increase in ^14^C ventilation leads the Atlantic by 2 to 3 ka, suggesting that most of the atmospheric CO_2_ increase during early Heinrich Stadial 1 (HS1) derives from carbon in the Southern and Pacific Oceans.

Not only does deep Atlantic ^14^C ventilation lag the Southern Ocean during the deglaciation, but the ^14^C age of Atlantic bottom water also increases during HS1 to become as old as the deep Pacific (Fig. 4B). We note that a prior compilation and examination of northwest Atlantic ^14^C/C did not capture this increase in ^14^C ventilation age during HS1 (*49*), likely due to the spatial limitation of their dataset. This marked increase in Atlantic bottom water ^14^C age during HS1 is best explained by a major reduction in NADW formation, which is corroborated by the correspondence between our North Atlantic ^14^C records, proxies for North Atlantic overturning (see increased ^231^Pa/^230^Th in Fig. 4C) (*50*), and reduced abyssal oxygenation (*51*). The HS1 reduction in NADW formation is generally attributed to a freshening of North Atlantic surface waters during HS1 (*50*), but Atlantic mid-depth ^14^C ventilation (influenced by upper NADW formation) is only minorly perturbed. This large difference in the response of Atlantic mid-depth and bottom water during HS1 could be explained by an increased sensitivity of lower NADW formation to this perturbation, allowing for Southern and Pacific bottom waters to have a greater influence on the ^14^C age of the deepest Atlantic waters.

The glacial-to-deglacial evolution of deep Pacific ventilation is different from either the deep Atlantic or Southern Ocean, with both mid-depth and bottom water ^14^C ventilation ages decreasing from ≈25 to 16 ka BP but maintaining a mid-depth to bottom water difference of +700 ± 150 years over this time period (Figs. 3F and 4B). We interpret this relatively static ^14^C age depth gradient in the Pacific as the persistence of the glacial Pacific overturning configuration, a continuation of the flipped Pacific even as (i) the ^14^C ages of both Pacific mid-depth and bottom waters decline by ≈400 years, (ii) atmospheric CO_2_ begins to rise (≈18 ka BP), and (iii) NADW formation is markedly reduced during early HS1 (18 to 16 ka BP). Thus, when the deep Pacific begins to reorganize from the glacial flipped to the modern configuration at ≈16 to 14 ka BP, there must have been a perturbation in addition to and/or greater than those listed above. One possibility is a change in the basin-scale freshwater balance, with saltier conditions and enhanced overturning in the Atlantic triggering a freshening and weakening of ventilation in the Pacific (*43*, *44*).

Both the ^14^C ventilation age and δ^13^C of glacial Pacific bottom waters suggest that glacial Pacific bottom water was carbon- and nutrient-rich; thus, their reexposure in the Southern Ocean surface predicts a larger-than-average supply of nutrient-rich, carbon-rich, and ^14^C-depleted waters. In late HS1, the ^14^C ages of Pacific and Atlantic mid-depth and bottom waters reduce toward their modern values, suggesting the reorganization of global ocean circulation toward the modern overturning configuration (Figs. 3 and 4B). This reintroduction of nutrient-rich waters to the Southern Ocean as part of the modern figure 8 overturning (Fig. 2A) during later HS1 may explain an anomalously high spike in Southern Ocean productivity (*52*) (Fig. 4D) and coincident steep decline in atmospheric Δ^14^C (Fig. 3C). This interpretation of the deep-sea ^14^C/C trends predicts that much of the carbon in the late HS1 increase in atmospheric CO_2_ derives from Pacific and Atlantic bottom waters.

The reorganization of global overturning from the glacial to the interglacial configuration appears to be fully accomplished by the Bølling-Allerød (BA; ≈14 ka BP) when deep Atlantic and Pacific ^14^C ventilation ages are not significantly different from today (Fig. 4). Among the observed variability during the BA is the “overshoot” of Southern Ocean mid-depth ^14^C ventilation ages during the BA (Figs. 3E and 4B) [as observed in (*17*, *53*)], which could be explained by an enhanced production of NADW alongside changes in upwelled water supply (*17*, *54*). Later, during the Younger Dryas (YD; 12.8 to 11.5 ka BP), when atmospheric Δ^14^C briefly increases for the only time during the deglaciation, we observe a complicated pattern in global deep-sea ^14^C ventilation age. There is a small increase in Atlantic bottom water ^14^C ventilation age that is not observed in Atlantic mid-depths (*55*)—a deep Atlantic ^14^C ventilation change that echoes the observations from the earlier, presumably freshwater-forced changes in Atlantic overturning during HS1. The steady decrease in Pacific Δ^14^C across the YD event (Fig. 3C) leads to a temporary increase in the ^14^C ventilation age during the YD (Fig. 3, C and F). Considering the brief (approximately hundreds of years) rise in atmospheric Δ^14^C, these patterns in deep Pacific ^14^C ventilation age may best reflect an insensitivity to a relatively short-lived change in atmospheric ^14^C/C after the last ice age.

Last, one alternate explanation for the deglacial rise in atmospheric CO_2_ is the input of geologic carbon associated with Pacific mid-ocean ridge volcanism (Fig. 4A) (*56*). This would increase the apparent deep-sea ^14^C ventilation ages by adding ^14^C-depleted carbon but is not supported with our dataset. Although some evidence exists for enhanced deglacial geologic carbon flux at shallower intermediate depths (*15*, *57*, *58*), the available datasets do not suggest that there was a large enough injection of ^14^C-depleted carbon to affect the basin-average values in Fig. 3 (*59*). Pacific mid-depth and bottom water ^14^C ventilation ages are younger during the first stage of the deglaciation than at any time during 25 ka BP (Figs. 3 and 4).

### Indo-Pacific Ocean glacial-interglacial dynamics

This work provides a synthesis of glacial-interglacial deep-sea ^14^C ventilation and that provides benchmarks for future studies of global ocean overturning strength and geometry. The deep Indian Ocean, where the number of glacial-interglacial measurements is not large enough to reliably produce basin-scale trends, is conspicuously absent and is a logical target for future work. The Indian and Pacific Oceans have been lumped together in past studies, but recent work suggests some major deviations in Indian deep-sea ^14^C ventilation (*60*). Whatever the case, the magnitude of the glacial-interglacial Pacific Ocean ^14^C ventilation changes shown in our study should widen the aperture of paleoceanographic research beyond ocean and atmospheric influences on the North Atlantic and Southern Ocean, to construct an integrated view of deep-sea dynamics since the last ice age.

## Supplementary Materials

### This PDF file includes:

Materials and Methods Figs. S1 to S10 Tables S1 and S2 References
