# Supplementary material for: Global reorganization of deep-sea circulation and carbon storage after the last ice age
Source: Sci Adv. 2022 Nov 16;8(46):eabq5434. doi: 10.1126/sciadv.abq5434 (PMC9668286; doi:10.1126/sciadv.abq5434)
Supplement: Supplementary file 1 — Materials and Methods Figs. S1 to S10 Tables S1 and S2 References [file sciadv.abq5434_sm.pdf]

Science Advances
AAAS

Supplementary Materials for

Global reorganization of deep-sea circulation and carbon storage after the last ice age

Patrick A. Rafter *et al.*

Corresponding author: Patrick A. Rafter, prafter@uci.edu

Sci. Adv. **8**, eabq5434 (2022)
DOI: 10.1126/sciadv.abq5434

This PDF file includes:

Materials and Methods
Figs. S1 to S10
Tables S1 and S2
References

Supplementary Materials for

Global reorganization of deep-sea circulation and carbon storage after the last ice age

Patrick A. Rafter 1, William R. Gray 2, Sophia K.V. Hines 3, Andrea Burke 4, Kassandra M. Costa 3, Julia Gottschalk 5, Mathis P. Hain 6, James W.B. Rae 4, John R. Southon 1, Maureen H. Walczak 7, Jimin Yu 8,9, Jess F. Adkins 10, Timothy DeVries 11

1. University of California Irvine, Irvine, CA, USA
2. Laboratoire des Science du Climat et de l'Environnement (LSCE/IPSL), Université-Paris-Saclay, Gif-sur-Yvette, France
3. Woods Hole Oceanographic Institution, Woods Hole, Massachusetts, USA
4. University of St. Andrews, St. Andrews, Scotland, UK
5. Institute of Geosciences, Kiel University, Kiel, Germany
6. University of California Santa Cruz, Santa Cruz, CA, USA
7. Oregon State University, Corvallis, OR, USA
8. Pilot National Laboratory for Marine Science and Technology (Qingdao), Qingdao, 266237, China
9. Australia National University, Canberra, AU
10. California Institute of Technology, Pasadena, CA, USA
11. Department of Geography and Earth Research Institute, University of California, Santa Barbara, CA, USA

*Corresponding author. Email: prafter@uci.edu

This PDF includes:
Materials and Methods
Figs. S1 to S10
Tables S1 to S2

Supplementary Materials and Methods

Reporting seawater and fossil radiocarbon concentrations ($^{14}C/C$)

The concentration of radiocarbon (^{14}C) in seawater can be expressed as $^{14}C/C$, reflecting the relationship between ^{14}C and total C atoms in seawater. We use this to refer to the concentration of radiocarbon in seawater, which is often described here as both delta notation and as an age referenced to 1950 (the BP in years BP). Delta notation corrects the $^{14}C/C$ for ^{13}C fractionation and decay since the year of collection (for seawater, this can be the year the measurement was made, but for fossils this would be the calendar age). Thus, the age model for sediment cores is critical for discussing any paleoceanographic data, but it is even more important when calculating $\Delta^{14}C$. Here is the equation for calculating $\Delta^{14}C$:

$$\Delta^{14}C = (\exp\text{^}(^{14}C\ age/-8033)) / (\exp\text{^}(calendar\ age / -8267)) - 1 * 1000$$

The 8033 is the average decay rate derived from the original "Libby half-life" of 5568 years (*62*), while the 8267 is the average decay rate for the more accurate half-life 5730± 40 years (*63*).

The ^{14}C age is calculated from the Fraction Modern or (Fm; the amount of ^{14}C relative to 1950, as measured in all radiocarbon laboratories) as:

^{14}C age = -8033 * LN(Fm)

Compiling the benthic foraminifera and deep-sea coral $^{14}C/C$ dataset
Seawater $^{14}C/C$ can be reconstructed using marine macro-fossils such as deep-sea corals, which are sporadically distributed both spatially and temporally, but can be dated with U-series measurements to provide an absolute calendar age (*64*). Marine microfossils of foraminifera are more widely distributed in space and time, but rely on a variety of other methods for determining the calendar age.

Building upon the published compilation from Zhao et al. (*21*), we searched the literature, as well as two known databases (NOAA's NGDC and PANGAEA) for all published benthic foraminifera and deep-sea coral $^{14}C/C$ data. Even though this study only deals with sites >=27.5 γ^n (neutral density in kg m^{-3}; see text), we have made the entire dataset (even for sites in adjacent seas and shallower than used in this study) available at unique DOI that cites every contributing author (i.e., all who have measured benthic foraminifera or deep-sea coral $^{14}C/C$). The dataset can be found at: doi.pangaea.de/10.1594/PANGAEA.946522. The location of all published and new deep-sea coral and foraminifera $^{14}C/C$ measurements is shown in Fig. 1A and the contribution of each compilation study is illustrated in Fig. S3.

Published proxy record age model methods
All published calendar ages (a.k.a., the "age model") were unchanged except for those based on planktic ^{14}C ages. Age models using planktic ^{14}C ages assume a surface ocean "reservoir age" (i.e., the offset from the contemporaneous atmospheric ^{14}C) and use an atmospheric $^{14}C/C$ reconstruction to identify the calendar age. Almost none of the published data in the compilation use the most recent atmospheric $^{14}C/C$ compilation (IntCal20) (*20*), so we calibrated all the planktic foraminifera ^{14}C-based age models to IntCal20 (see below).

Updating the planktic ^{14}C-based age models. The site of each proxy record using planktic ^{14}C measurements to construct their age model undoubtedly has a unique reservoir age for each planktic ^{14}C measurement—reflecting both local circulation and the different air-sea equilibrium timescales for the isotopes (*31*, *32*)—but the IntCal community has provided an estimated reservoir age (the Marine20 calibration curve (*65*)) that is applicable to sites ±40° of the equator. For sites poleward of 40°N and 40°S, we have added an additional reservoir age based on the original publication. The reservoir age for the new sites outside of ±40° used the LGM mean and LGM error from (*66*). All planktic foram ^{14}C-based age models were updated with the R programming package Bacon (*67*), which uses Bayesian statistics to give mean values as well as errors. A sensitivity test of our reservoir age estimates is shown in Fig. S9, where we assumed an additional +1000-year reservoir age for all sites poleward of the 40° latitude. When more than one planktic species ^{14}C age was

available, the more "robust" (i.e., less prone to dissolution) species were used to construct the age model (as recommended in (*68*, *69*)). However, using the primary datasets found in (*70*, *71*), it was often the case that these robust microfossils have ^{14}C ages that were *older* than the benthic foraminifera ^{14}C ages during the Holocene. In these cases, the mean planktic ^{14}C age was used to construct the age model. The code for updating all the planktic ^{14}C-based age models in our new compilation can be found at: 10.5281/zenodo.7112182. Note that there is a column in the compilation ("Delta.R") for modifying the surface water ^{14}C reservoir age in addition to Marine20 (*65*) assumptions.

New radiocarbon analyses

Sediment from subarctic North Pacific, central North Pacific, and Subantarctic sites (see Fig. S4 and detailed site descriptions below) was washed using deionized water in a 63 μm sieve, and mixed benthic foraminifera species (notably without *Pyrgo* spp. (see (*72*, *73*) for more information) were selected from the >250 μm fraction. At least 10% of each sample was dissolved using HCl to remove potential secondary calcite (precipitated post-deposition). Samples were graphitized following (*74*) and analyzed at the Keck Carbon Cycle Accelerator Mass Spectrometry Laboratory at University of California, Irvine (*75*). All new sample measurements and previously published are available at: doi.pangaea.de/10.1594/PANGAEA.946522.

Stable isotopic composition of carbon analyses

Samples from Juan de Fuca Ridge sites (see detailed text below) were freeze-dried, and then an aliquot was weighed into a 125 mL Nalgene bottle, filled with approximately 100 mL of tap water, and disaggregated on a tumble wheel for two hours. Samples were then sieved at 63μm, and a soft brush was used to gently break up clay clumps that did not sufficiently disintegrate during tumbling. *Planulina wuellerstorfi* tests were picked from the 250-300um fraction and analyzed at Lamont Doherty Earth Observatory (LDEO) on a Thermo-Delta V Plus equipped with a Kiel IV individual acid bath device. The stable carbon isotopic ratio is reported in delta notation, where:

$$\delta^{13}\text{C} = \left(\frac{\frac{13C}{12C}sample}{\frac{13C}{12C}standard}\right) - 1,$$

where the standard is VPDB. This is multiplied by 1000 to give "per mil." Samples have been standardized to NBS-19, and all data are reported on the VPDB scale with precision ≤0.05‰.

Modern seawater $^{14}C/C$ statistics

Nearly all modern seawater $^{14}C/C$ measurements have taken place since the introduction of 'bomb' $^{14}C/C$—via the atmospheric testing of thermonuclear weapons—and are therefore susceptible to being biased toward artificially high $^{14}C/C$ and therefore young ^{14}C ages (*30*, *76*). While some data products attempt to remove this 'bomb' $^{14}C/C$ influence from seawater measurements (*77*), the use of this "natural" $^{14}C/C$ data product has been questioned (i.e., see comparison and discussion in (*78*)). We do not use the "natural" seawater $^{14}C/C$ data product, but also note that it also does not appear to be publicly

available. Instead, in Fig. 1B and Fig. S1 we have filtered the available seawater $^{14}C/C$ data to remove measurements made since bomb $^{14}C/C$ was introduced to the Earth system. This is not ideal since it obviously lowers the number of usable observations, but we find it to be the most conservative approach.

To avoid the influence of bomb $^{14}C/C$, we first removed seawater samples with a $\Delta^{14}C > 0$ ‰, effectively excluding measurements that are obviously influenced by bomb ^{14}C. To exclude seawater biased to higher $\Delta^{14}C$ by 'bomb' $^{14}C/C$, but still below 0‰, we further filter the seawater $^{14}C/C$ data to exclude samples with an CFC-11 > 0.1 pmol kg^{-1}. Our rationale for this value is based on simple calculations (see https://water.usgs.gov/lab/software/USGS_CFC/) showing that a seawater CFC-11 concentration of 0.1 pmol kg^{-1} is equivalent to waters that equilibrated with the atmosphere at ≈1955, which is one of the last years before the 'bomb' spike in atmospheric $^{14}C/C$ (*76*). Where there was no observed DIC ^{14}C age measurement within a reasonable depth and lateral distance from the proxy site (±500 m and ±5° of longitude and latitude), we used the $^{14}C/C$ from the Ocean Circulation Inverse Model (*79*).

Testing the proxy ^{14}C fidelity
We identify the utility of the global compilation for reconstructing past changes in seawater $^{14}C/C$ in Fig. 1B and Fig. S1. This test compares modern seawater $^{14}C/C$ with all proxy $^{14}C/C$ over the past 4-kyr BP and gives a relationship of $Y = (0.97\pm0.1)X + (-18\pm91)$; $R^2=0.66$. The strong correlation, slope close to 1, and Y intercept close to zero in Fig. 1B suggests that the marine fossil $^{14}C/C$ performs well as a proxy for seawater DIC $^{14}C/C$. Additional examinations of the proxy vs. seawater ^{14}C /C relationship over different time-intervals can be found in Fig. S1, all showing a similar, strong relationship (see figure and caption for detailed statistics). These age boundary tests (Fig. S1) suggest variability of the Y-intercept from -44-to- +54 ^{14}C years, which should be considered a minimum error for the marine ^{14}C proxy. Seawater $^{14}C/C$ deviations from modern values could explain some of the variance in Fig. 1B, but given the individual offsets from the 1:1 line of >100 years, the proxy itself is almost certainly the first-order driver of the variability (consider the findings in (*80*, *81*)). However, it is important to note that the potential bias (given by the Y-intercept) or the individual measurement deviations from the 1:1 line are much smaller than some reported ^{14}C biases of >10,000 ^{14}C years (*82*). As such, we argue that—despite known influences on the proxy ^{14}C archives from sedimentary geochemical processes (*80*), sedimentary bioturbation, and calendar age assumptions (*83*)—the results of our tests speak to the utility of the seawater $^{14}C/C$ proxy, especially interpretations based on a relatively large collection of measurements.

Pre-treating the benthic $^{14}C/C$ dataset
The data shown throughout the manuscript (e.g., Fig. 2, 3, & 4) was pre-treated to remove outlier observations in the following way. First, $^{14}C/C$ values that were above the contemporaneous atmosphere were flagged as outliers and not included. Second, $^{14}C/C$ values more than 3 sigma of the 500-year binned average (see below) were flagged as outliers (same as in (*33*)). We iterated this second outlier identification process (identifying a new binned mean and binned standard deviation plus removing the >3 sigma outliers) four times to ensure outlier removal. The outlier data points are clearly noted in the compilation file and can also be observed as pink diamonds in Fig. S5-to-S8. Note that

500-year bins with only 1 measurement were also considered "outliers", are not used in the LOESS trend estimate (see below)—these are also shown as pink diamonds in Fig. S5-to-S8.

Density surfaces for mid-depth and bottom waters

We base our interpretation of past deep-sea circulation on modern deep-sea circulation, which can be defined by neutral density surfaces. The "mid-depth" waters include modern Pacific Deep Water (PDW) and Upper North Atlantic Deep Water (NADW) and are within the neutral density surfaces of 27.5 and 28.0 kg m^{-3} (*23*, *84*). The denser "Bottom Waters" (>28.0 kg m^{-3}) include Lower North Atlantic Deep Water and Antarctic Bottom Water (AABW).

Proxy data trend and uncertainty estimates

We pull out the basin wide trends (and the associated uncertainties) from the data using two independent methods: binning and LOESS regression**.**

Proxy $^{14}C/C$ measurements were binned in 500-year intervals (based on calendar age). This follows prior arguments that the binning width should reflect the mean calendar age error (*21*). The average calendar year error of the compilation is ±250 years, but this does not take into account various assumptions including changes in surface ocean ^{14}C reservoir ages (used in planktic ^{14}C-based age models), errors associate with the different age model assumptions (*6*), and more. To account for these additional, unknown errors, we assumed a binning width of 500-years. After subsetting the data at 500-year time steps, we remove the three sigma outliers (pink diamonds in Fig. S5-to-S8) and calculate the mean, standard deviation, and standard error of each bin. The mean bin values are shown as black symbols in Fig. S5-to-S8 and the standard error is shown by vertical lines. These binned values are different than the LOESS estimated $^{14}C/C$ trends.

Fossil ^{14}C data were fit with a non-parametric regression (LOESS, a locally estimated smoothing) as a function of calendar age in R (R core team), with the smoothing parameter (α) optimized by Generalized Cross Validation (GCV). Note, the LOESS regressions are fit to the pre-treated (see above), unbinned ^{14}C data (with 3-sigma and other outliers removed). The most likely fit to the ^{14}C data and uncertainties were calculated with a Monte-Carlo approach (1000 iterations); this includes a bootstrap resampling of the dataset (*85*), and Monte-Carlo resampling of the calendar age and ^{14}C analytical errors, as well as propagation of these errors through to the $\Delta^{14}C$ and ventilation age estimates in a manner which accounts for the covariance of the calendar age uncertainties with the $\Delta^{14}C$ and ventilation age uncertainties. We report the uncertainties as the 5th, 32nd, 68th, and 95th percentiles of these LOESS fits (with the best fit taken as the 50th percentile), including a Monte-Carlo resampling of the SE of the LOESS fit during each iteration.

The code for creating the basin mean LOESS trends for ^{14}C ventilation age can be found at: https://github.com/patrickrafter1/14C-compilation-2022.

Site bias adjustments to calculate the basin mean $^{14}C/C$

Note that using relatively sparse sites for reconstructing a basin mean could be biased to sites that have a higher or lower value than the actual basin mean for those density

surfaces (see above). In consideration of this potential biasing, we adjust each record based on the difference between the modern seawater ^{14}C bathing the site and the modern mean value for that basin. In other words, each fossil ^{14}C measurement was adjusted to account for seawater $^{14}C/C$ differences between the basin average and the proxy record location. It is this "site bias corrected" data that was used to construct the trends in Fig. 3 & Fig. 4. These same trends, binned mean values (black circles for mid-depth and black squares for bottom waters), binned standard error (lines), all accepted observations (circles, color-coded to original compilation), and outlier data points (pink diamonds) can be observed in Fig. S5 & S6. Note that a first-order test of this approach can be observed by the approximate overlap between most late Holocene reconstructed and modern observed $^{14}C/C$ (symbols to the left in all figures).

We also show the "without site bias correction" trends and datapoints in Fig. S7 (shown as the uncorrected $\Delta^{14}C$) & Fig. S8 (shown as the uncorrected ^{14}C ventilation age). These "uncorrected" trends show that the adjustments do not make significant changes to the reconstructions, although they slightly increase the standard error and worsen the overlap between the trend and the observed, ship-based dissolved inorganic carbon (DIC) $^{14}C/C$ (symbols in Fig. S5-to-S8). One notable difference is for the Southern mid-depth $^{14}C/C$, which is without the site/ depth bias correction gives a younger age than the observed modern value (circle to the left in each plot).

Sensitivity testing the basin mean ^{14}C ventilation age trends

In Fig. S9 & Fig. S10, we test the sensitivity of the assumptions used in creating the ^{14}C ventilation trends in Fig. 3 & Fig. 4. The site-bias / depth-bias corrections with our assumed depth surface separations are shown in panels (A) to (C) in both figures (see text above for details and rationale for this approach). Fig. S9 (D-to-F) shows the basin-averaged ^{14}C ventilation trends without the depth-bias correction. In Fig. S9 (G-to-I), we show the ^{14}C ventilation trends assuming that an additional 1000-year reservoir age at sites poleward of 40°N and 40°S may be a better assumption for planktic foraminifera ^{14}C-based age models. Another concern when using benthic foraminifera $^{14}C/C$ measurements is that bioturbation may influence the observed ^{14}C age and in Fig. S9 (J-to-L), we create basin-averaged ^{14}C ventilation trends only using sediment cores with sedimentation rates >2 cm kyr^{-1}. In Fig. S10, we provide additional sensitivity tests assuming different density surfaces: (A-to-C) uses our default 27.5-to-28 kg m^{-3} and >28 kg m^{-3} density surfaces; (D-to-F) uses the 27.4-to-28 kg m^{-3} and >28 kg m^{-3} density surfaces; (G-to-I) uses the 27.6-to-28 kg m^{-3} and >28 kg m^{-3} density surfaces; and (J-to-L) uses the 27.5-to-28.1 kg m^{-3} and >28.1 kg m^{-3} density surfaces.

While these different assumptions adjust the finer details of the estimated basin-scale ^{14}C ventilation age, it appears that our main ^{14}C ventilation age discussion points—older ^{14}C ages of all bottom waters, the inversion of Pacific mid-depth and bottom water ages, the different LGM variability in all basins—are still supported by these varied sensitivity tests.

T-tests of the flipped Pacific ^{14}C ventilation ages

A simple t-test of our results lends further support for a significant difference between Pacific mid-depth and bottom water ^{14}C ventilation ages during the LGM, for both our

default case and these sensitivity tests. For example, the estimated Pacific trends from our default assumptions (Fig. S9 Panels A-to-C) indicate there is a significant difference between Pacific mid-depth and bottom water ^{14}C ventilation ages from 23-to-18-kyr BP (t-test results: t = -4.8345, p-value = 2.28e-06). Contrast this with a similar analysis of Pacific mid-depth and bottom water ^{14}C ventilation ages from 6-to-0-kyr BP indicating no significant difference (Welch's t-test results: t = 0.13002, p-value = 0.8968). Looking to one of the more different results of our sensitivity tests, the estimated Pacific ^{14}C/C from the "+1000-year reservoir age sensitivity test" (Fig. S9; panels G-to-I) also indicates a significant difference between Pacific mid-depth and bottom water ^{14}C ventilation age during the LGM (t = -2.9036, p-value = 0.004031), but no significant difference during the late Holocene (t = 0.32647, p-value = 0.7449).

Estimating average bottom water ^{14}C/C during the LGM

The volume of Atlantic, Southern, and Pacific mid-depth and bottom waters was estimated from the Ocean Circulation Inverse Model (OCIM) (*79*), assuming glacial changes in volume are negligible for these calculations. Note that the Indian Ocean contains so few values that it was not used in this calculation. The ^{14}C ventilation age during the LGM and percent of global ocean volume for each ocean basin and density range are as follows. Mid-depths: Atlantic bottom waters are estimated to be assumed to be 22%, the Southern Ocean is assumed to be 16%, and the Pacific is assumed to be 46% of global waters along these density surfaces. Bottom waters: Atlantic waters are assumed to be 19%, the Southern Ocean is assumed to be 21%, and the Pacific is assumed to be 46% of global waters along these density surfaces. The global mid-depth and bottom water ^{14}C ventilation age was estimated for each 500-year bin over the past 25-kyr using LOESS (see above) and this time-interval was weighted by the ocean basin volume percent. The average and standard error for relevant time-intervals are shown in Supplementary Tables 1 and 2, along with their difference from modern DIC ^{14}C ventilation age (see above).

Differences in LGM time-slice depth profile and global average ^{14}C ventilation estimates

We used our ^{14}C/C compilation to reconstruct the ^{14}C ventilation age of the Pacific with depth during the LGM in Fig. S2 (panel C) and this "time-slice" view of the dataset is one piece of evidence—along with an LGM time-slice of δ^{13}C and the full glacial-interglacial time-series in Fig. 3, Fig. 4, Fig. S3-to-S8—indicating that the oldest ^{14}C ages are found in the deepest Pacific Ocean during the LGM. However, we only have one observation for the Pacific Ocean >4000 m (see our Fig. 2D & Fig. S2), which differs from earlier work (*33*) including 6 "Pacific / Indian" observations >4000 m (their Figure 3B). Breaking down the data used to construct the LGM Pacific time-slice in (*33*) (using their compilation available online), their Figure 3B below 4000 m includes 1 measurement from the Central Equatorial Pacific and 5 measurements that are south of 40°S (off of southern New Zealand). Of these observations, only the Central Equatorial Pacific measurement falls within our definition of the Pacific Ocean (between 40°S and 60°N). Thus, the difference in LGM Pacific depth-profile of ^{14}C ventilation age shown here (Fig. S2) and in prior work (Figure 3B in (*33*)) is caused by our geographic definition for the Southern Ocean (see Fig. 1A). Considering the much larger volume of the Pacific Ocean, our decision not to include these Southern New

Zealand observations likely contributed to our significantly older estimated global average ^{14}C ventilation age than in prior work (*6*, *33*).

New proxy ^{14}C/C measurements from the Pacific Ocean
We have generated several new records of mixed benthic foraminifera ^{14}C/C from the Pacific Ocean and extended a recently published mixed benthic foram record (*86*) from a site in the Southern Ocean (see Fig. 1A). No *Pyrgo* spp. were included in these measurements per earlier studies (*72*, *73*). All new measurements were measured in the Keck Carbon Cycle AMS Laboratory at UC Irvine according to the preparation and measurement protocols found in (*74*, *75*). The results of our new measurements (and comparisons with trends from all sites within that seawater density range) can be seen in Fig. S7. Below we provide additional details for each site.

Southern Ocean site MD97-2106. New benthic foraminifera ^{14}C measurements from Southern Ocean site MD97-2106 (45.2°S, 146.3°E; 3310 m water depth) were added to published results (*86*) to extend the record back to 25-kyr BP. The age model for these new measurements is based on planktic ^{14}C ages on the Marine20 calibration with additional reservoir age of 256±219 years, derived from average values for that time and location published in (*66*). Among the new insights the core brings is the decrease in ^{14}C ventilation age during HS2 as seen in another Southern Ocean Bottom-Water record (MD07-3076 at 3770 m water depth) in the Atlantic Sector of the Southern Ocean (*5*).

North Pacific site VINO19-4 GGC-37. We also have new benthic foraminifera ^{14}C measurements from Subarctic Pacific site VINO19-4 GGC-37 (50.4°N, 167.7°E; 3300 m water depth). The age model here uses a combination of stratigraphic matching and planktic ^{14}C ages calibrated using Marine20. We applied an additional reservoir age correction of 400±400 years applied to the Marine20 because of the core's higher latitude (see above). One stratigraphic tie-point is used where the large decline in planktic δ^{18}O (see original publication (*35*)) is tied to the onset of the BA/ACR at 14.7-kyr BP. The two Δ^{14}C spikes to high values observed during the middle of the BA/ACR and ≈10-kyr BP are removed as 2-sigma outliers, although earlier studies have suggested brief, but deep penetration of surface waters in the Subarctic Pacific (*43*). Regardless, the high resolution of our measurements diminishes the importance of these two seemingly-anomalous data points

Tropical Pacific site ML1208-01PC. Our third new benthic foraminifera ^{14}C/C record is from site ML1208-01PC (21.2°N, 158.5°W; 2960 m water depth). The age model for this sediment core west of Hawai'i was constructed using a combination of stratigraphic matching (increasing sediment density change beginning at 23-24 cm was assumed to be 18-kyr BP) and planktic ^{14}C-based age model calibration (to MARINE20) at planktic foraminifera abundance maxima. The benthic foraminifera ^{14}C ventilation age has a larger degree of variability around the Pacific Bottom-Water trend (blue line), which likely speaks to the impacts of bioturbation on this relatively slowly accumulating sediment core.

Figures and other acknowledgments. Some figures were initially generated using Ocean Data View (*87*) and utilize color palettes developed by (*88*).

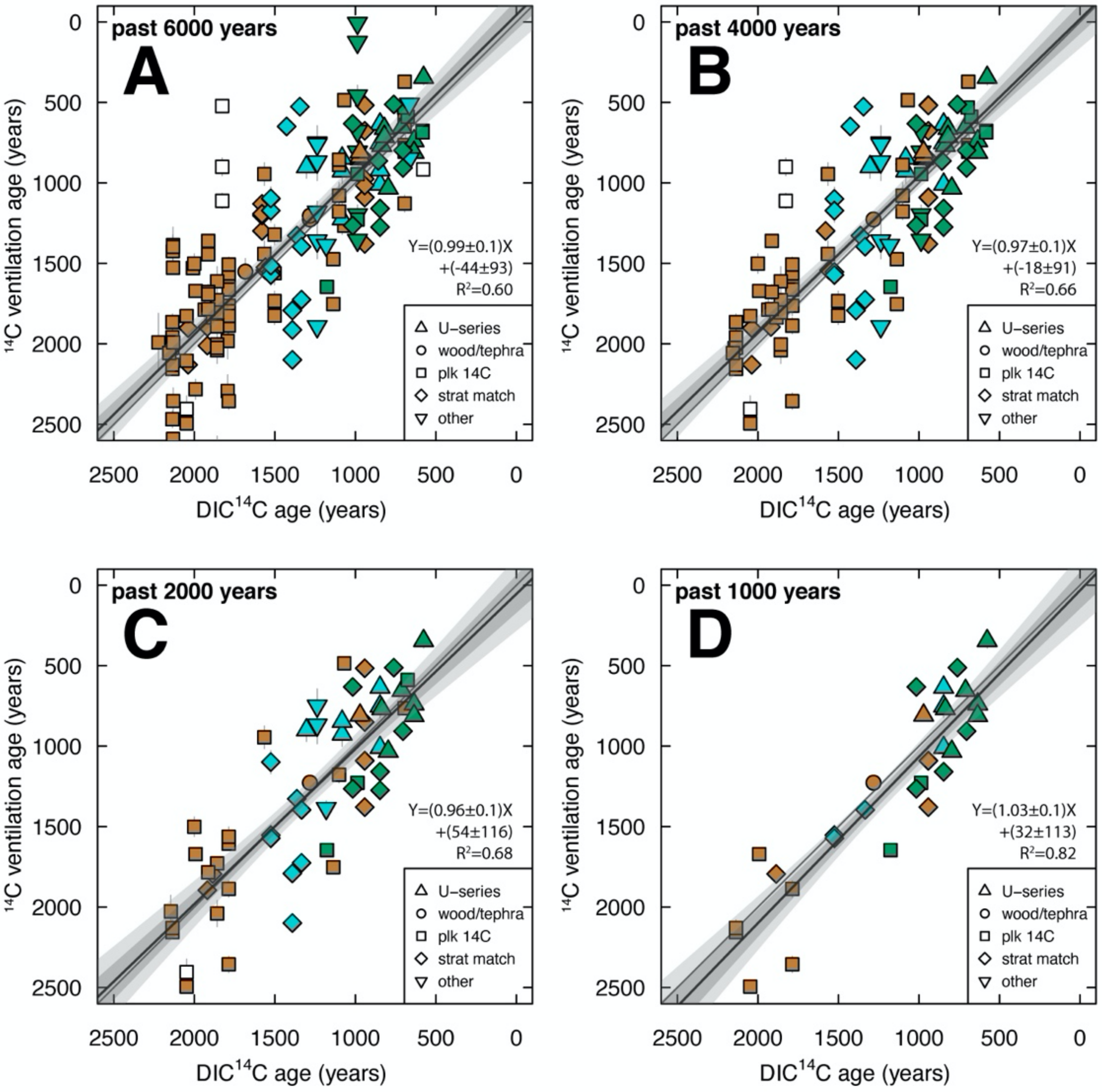

Figure S1: Examining modern and marine fossil late Holocene fossil $^{14}C/C$. The x axis in all plots is the modern DIC $\Delta^{14}C$ at the proxy site and the Y axis is the difference between the contemporaneous atmosphere and fossil ^{14}C ("^{14}C ventilation age") for varying calendar age boundaries. Shaded regions are 1 and 2 sigma confidence intervals for linear regression. Color shading is the ocean basin of the proxy measurement (green is Atlantic, light blue is Southern, and brown is Pacific; see Fig. 1A for legend). The statistics for these analyses are as follows: (A) Measurements from the last 6-kyr BP: Y = (0.99±0.1)X + (-44±93); R^2 = 0.60. (B) Measurements from the last 4-kyr BP: Y = (0.97±0.1)X + (-18±91); R^2 = 0.66. (C) Measurements from the last 2-kyr BP: Y = (0.96±0.1)X + (54±116); R^2 = 0.68. (D) Measurements from the last 1-kyr BP: Y = (1.03±0.1)X + (32±113); R^2 = 0.82. The symbols in the legend signify the age model used for the study: Triangles for deep-sea coral and U-series dates; Circles for wood or tephra dating; Square for planktic foraminifera ^{14}C calibrated to the atmosphere; Diamonds for a variety of stratigraphic matching (e.g., oxygen isotopes matched to deep-sea compilations); and upside-down triangles show other methods (e.g., plateau-tuning of $^{14}C/C$).

Table S1: The volume-weighted mid-depth water (between the 27.5 and 28 kg m^{-3} neutral density surfaces) ^{14}C ventilation ages is slightly older during the LGM and are within error of modern values only mid-way through the deglacial global warming. Modern $^{14}C/C$ observations are from (*30*) and are rounded to the 10. Reconstructed ^{14}C ventilation values are rounded to the 50. The difference between modern and reconstructed ^{14}C ventilation ages and their propagated errors are also shown (right side).

volume-weighted average ^{14}C ventilation age for Atlantic, Southern, and Pacific Ocean mid-depth water	^{14}C ventilation age (years)	^{14}C ventilation age standard deviation (years)	^{14}C ventilation age difference	^{14}C ventilation age difference (propagated standard deviation)
PRE-INDUSTRIAL (observed)	1290	110	0	--
HOLOCENE (10-to-0-kyr BP)	1300	150	0	150
YOUNGER DRYAS (12.8-to-10-kyr BP)	1250	100	-50	150
BA/ACR (14.7-to-12.8-kyr BP)	1300	100	0	150
HS1 (18-to-14.7-kyr BP)	1500	100	200	150
LGM (23-to-18-kyr BP)	1750	150	450	150

Table S2: The volume-weighted bottom water (below the 28 kg m^{-3} neutral density surface) ^{14}C ventilation ages is much older during the LGM and within error of modern values mid-way through the deglacial global warming. Here, Atlantic waters are assumed to be 19%, the Southern Ocean is assumed to be 21%, and the Pacific is assumed to be 46% of global waters along these density surfaces. Modern $^{14}C/C$ observations are from (*30*) and are rounded to the 10. Reconstructed ^{14}C ventilation values are rounded to the 50. The difference between modern and reconstructed ^{14}C ventilation ages and their propagated errors are also shown (right side).

volume-weighted average ^{14}C ventilation age for Atlantic, Southern, and Pacific Ocean bottom water	^{14}C ventilation age (years)	^{14}C ventilation age standard error (years)	^{14}C ventilation age difference	^{14}C ventilation age difference (propagated standard deviation)
PRE-INDUSTRIAL (observed)	1350	90	0	--
HOLOCENE (10-to-0-kyr BP)	1300	150	-50	150
YOUNGER DRYAS (12.8-to-10-kyr BP)	1500	150	150	150
BA/ACR (14.7-to-12.8-kyr BP)	1450	150	100	150
HS1 (18-to-14.7-kyr BP)	2050	150	700	150
LGM (23-to-18-kyr BP)	2350	150	1050	150

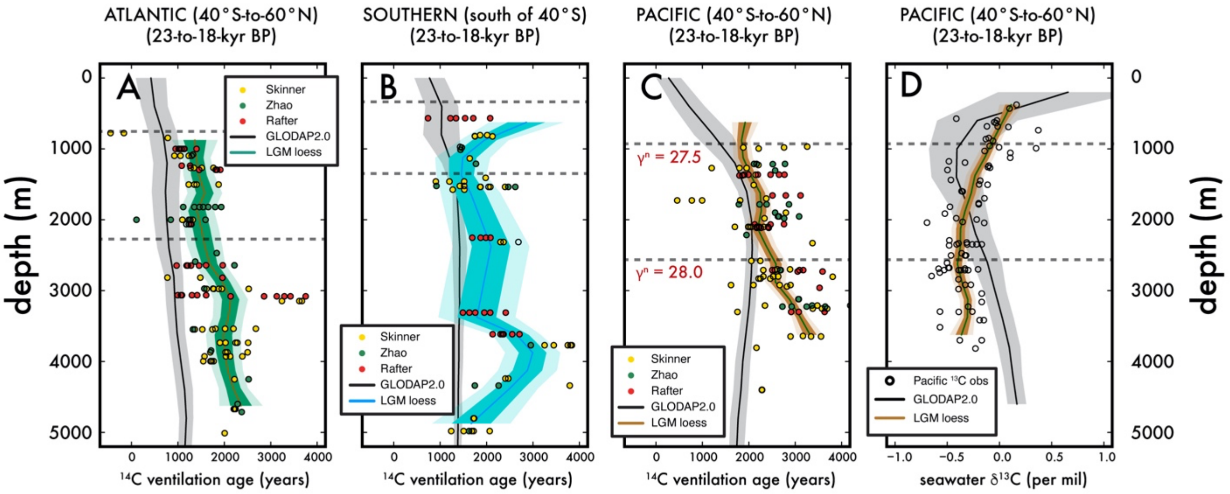

Figure S2: Depth-binned LGM seawater ^{14}C ventilation age (colors) is significantly older than pre-industrial seawater carbon isotopic composition (black). The pre-industrial average seawater ^{14}C ventilation ages with depth for (A) Atlantic, (B) Southern, and (C) Pacific Oceans are shown by the vertical black lines, where the grey envelope is 1-standard error (data from the GLODAP v.2 (*30*)). Colored lines show average seawater ^{14}C ventilation ages with depth during the LGM (23-to-18-kyr BP). Circles in (A)-to-(C) are LGM proxy observations (23-to-18-kyr BP). The different color envelopes are 1- and 2-Standard Error. We color-code the LGM observations based on their compilation study (see Legend). In (D), the pre-industrial average seawater $\delta^{13}C$ with depth for the Pacific (GLODAP v.2; black with grey envelope) is compared with new and newly-compiled North Pacific benthic foraminifera $\delta^{13}C$ from the LGM (brown line with 1 and 2 Standard Error envelopes) (*35*–*37*). The upper and lower dashed lines in all figures represent the modern regional mean depth of the 27.5 and 28.0 neutral density surfaces, respectively (*89*). See Supplementary Material (SM) for benthic foram $\delta^{13}C$ methods. Basin average trends with depth were estimated using LOESS (see SM for methods).

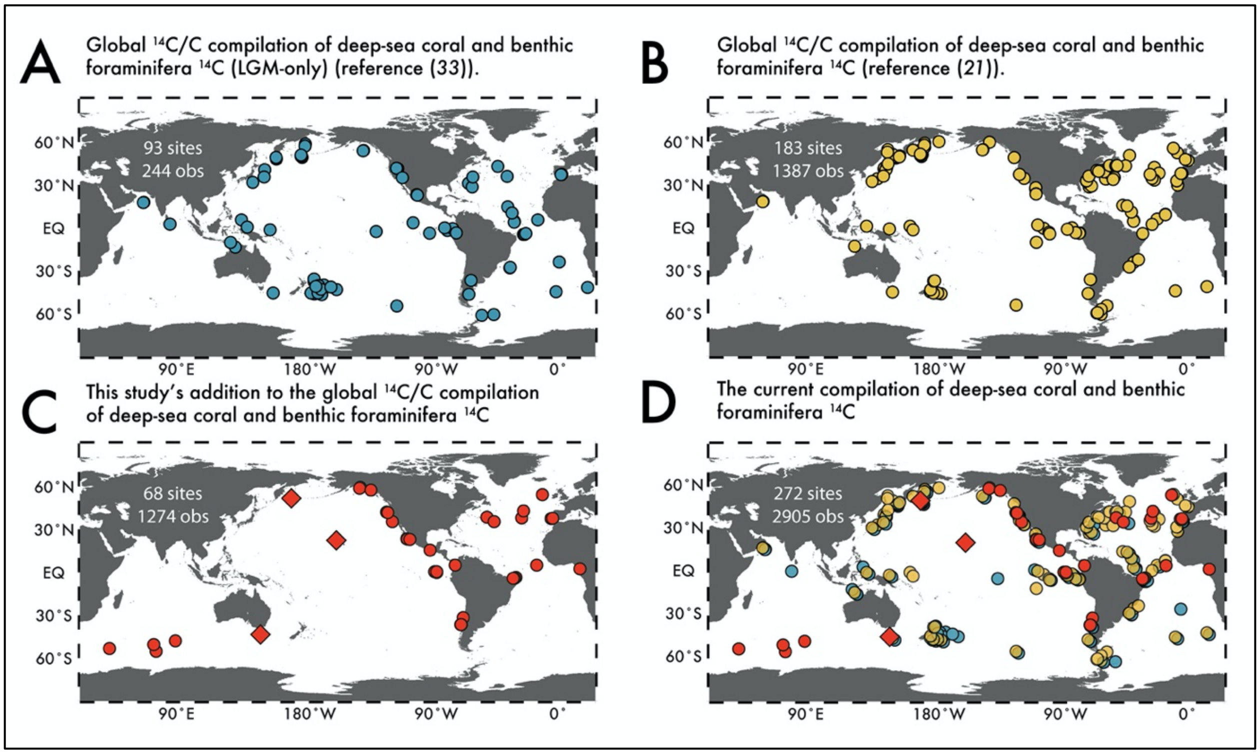

Figure S3: The spatial distribution for the different benthic foraminifera and deep-sea coral $^{14}C/C$ compilations. Sites in the reference (*33*) compilation are shown in (A) and sites in the reference (*21*) compilation are shown in (B). There is some overlap in the location of the reference (*33*) compilation sites (A) and the reference (*21*) compilation sites because only LGM-aged observations were included in (A). In addition to creating 3 new glacial-interglacial $^{14}C/C$ records (diamonds here and in Figure 1), our new compilation includes the sites shown in (C). All available $^{14}C/C$ observations are shown in (D). The number of unique sites and observations for each compilation is shown in each plot. Different compilation studies report observations from the same site, so that the sum of the unique sites in panels A-to-C do not equal the unique sites in Panel D.

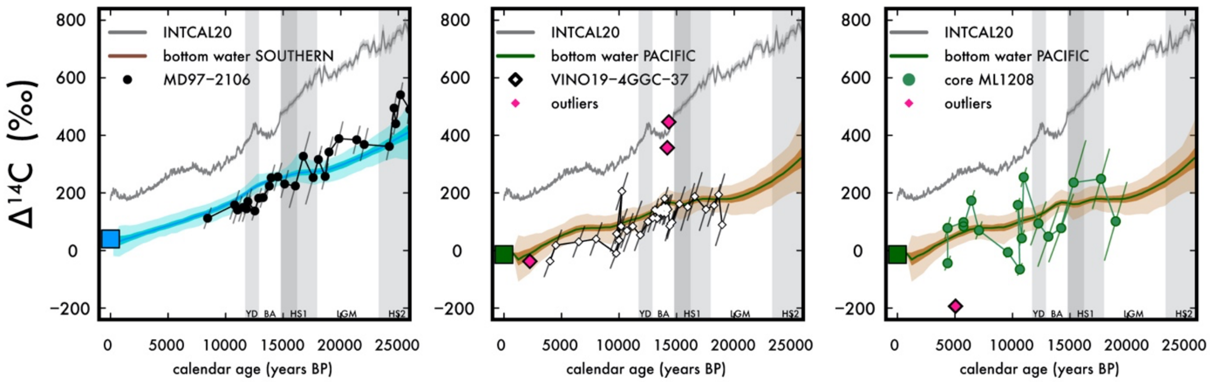

Figure S4. New $^{14}C/C$ measurements on cores from the Pacific and Southern Oceans. The $\Delta^{14}C$ for three new sites in: (left) the Southern Ocean (MD97-2106; -45.2°N, 146.3°E; 3310 m), (center) the Subarctic Pacific (VINO 19-4 GGC-37; 50.4°N, 167.7°E; 3300 m), and (right) Subtropical North Pacific (ML1208; 21.2°N, 158.5°W; 2960 m). Each is compared to atmospheric $\Delta^{14}C$ (grey line (*5*)) and LOESS-averaged deep water $\Delta^{14}C$ for each basin (neutral density (γ^n) >28; see Fig. 3 and main text). Symbols to left are the average modern seawater $\Delta^{14}C$ values for each ocean basin with γ^n >28 kg m^{-3}. Pink diamonds are not used to create the basin averages, either having a value that was >3 standard-deviation units of the binned mean value or the only value within that 500-year bin. HS2= Heinrich Stadial 2; LGM=Last Glacial Maximum; HS1=Heinrich Stadial 1; BA=Bølling-Allerød; YD = Younger Dryas.

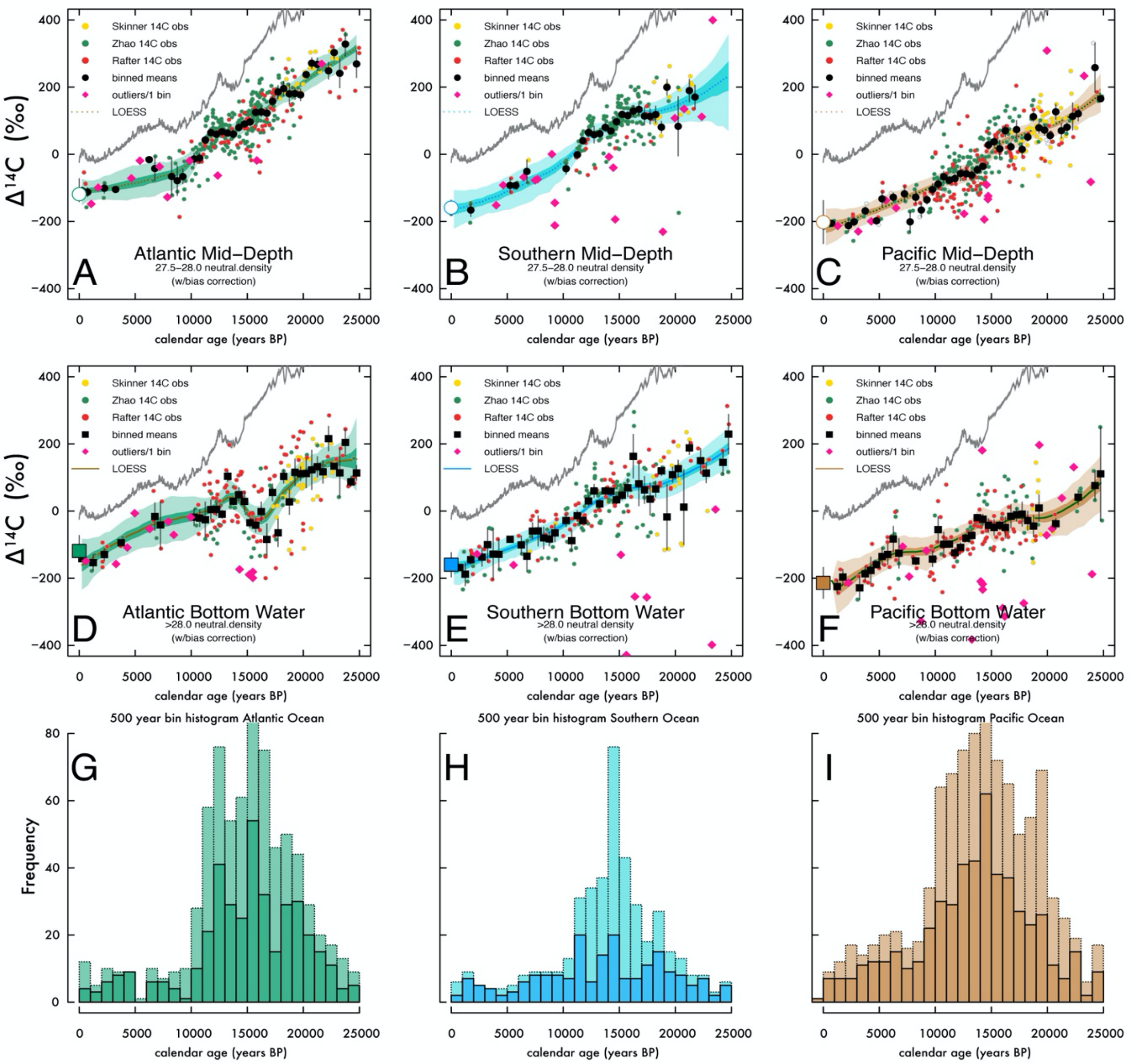

Figure S5: Mean seawater $\Delta^{14}C$ trends for Atlantic, Southern, and Pacific Oceans (left-to-right) with site bias correction. Here we show the LOESS trends of $\Delta^{14}C$ (in per mil) from Fig. 3 plus all the observations (grey circles), sorted by their published compilation (see legend). Black symbols and lines are binned mean values and 1-standard error, respectively. Pink diamonds are observations that are >3-standard deviation the binned mean or bins with only one observation (note that these are not used in the LOESS estimate). Panels (A-C) are for mid-depth density surfaces and panels (D-F) are for bottom water density surfaces. Panels (G-I) are the histograms for the observations used to create the LOESS trends, dotted outline bars are mid-depth and solid outline bars are bottom-waters. The depth-bias correction involves an adjustment applied to each observation in consideration of site biasing relative to the basin average for that depth range (see Methods). LOESS error envelopes show 68% and 95% confidence interval from bootstrapping/Monte-Carlo simulation (see Methods)

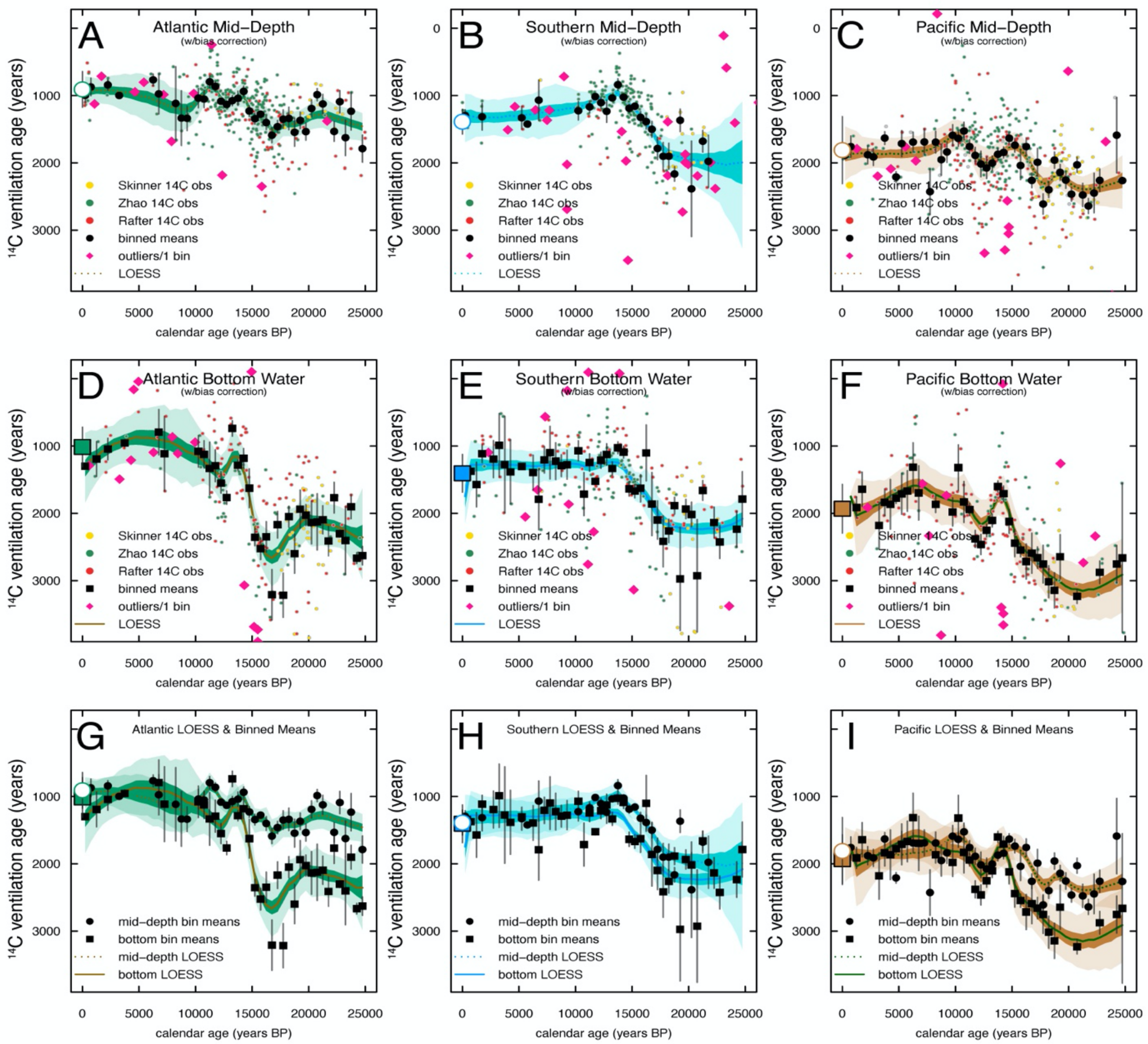

Figure S6: Trends and binned mean values for ^{14}C ventilation age in the Atlantic, Southern, and Pacific Oceans (with site bias correction). Here we show the LOESS trends of the ^{14}C ventilation age (years) from Fig. 3 plus all the observations (grey circles). Black symbols and lines show binned mean values used for calculating the LOESS trends and 1-standard error, respectively. Pink diamonds are observations that are >3-standard deviation the binned mean or bins with only one observation (these outliers are not used in the LOESS estimate). Panels (A-C) are for mid-depth density surfaces and panels (D-F) are for bottom water density surfaces. Panels (G-I) show all the data together and is included to illustrate the ^{14}C ventilation age differences between mid-depth and bottom waters, regardless of analysis. In all figures, dashed lines and circles are mid-depth and solid lines and squares are bottom waters. The depth-bias correction involves an adjustment applied to each observation in consideration of site biasing relative to the basin average for that depth range (see Methods). LOESS error envelopes show 68% and 95% confidence interval from bootstrapping/Monte-Carlo simulation (see Methods).

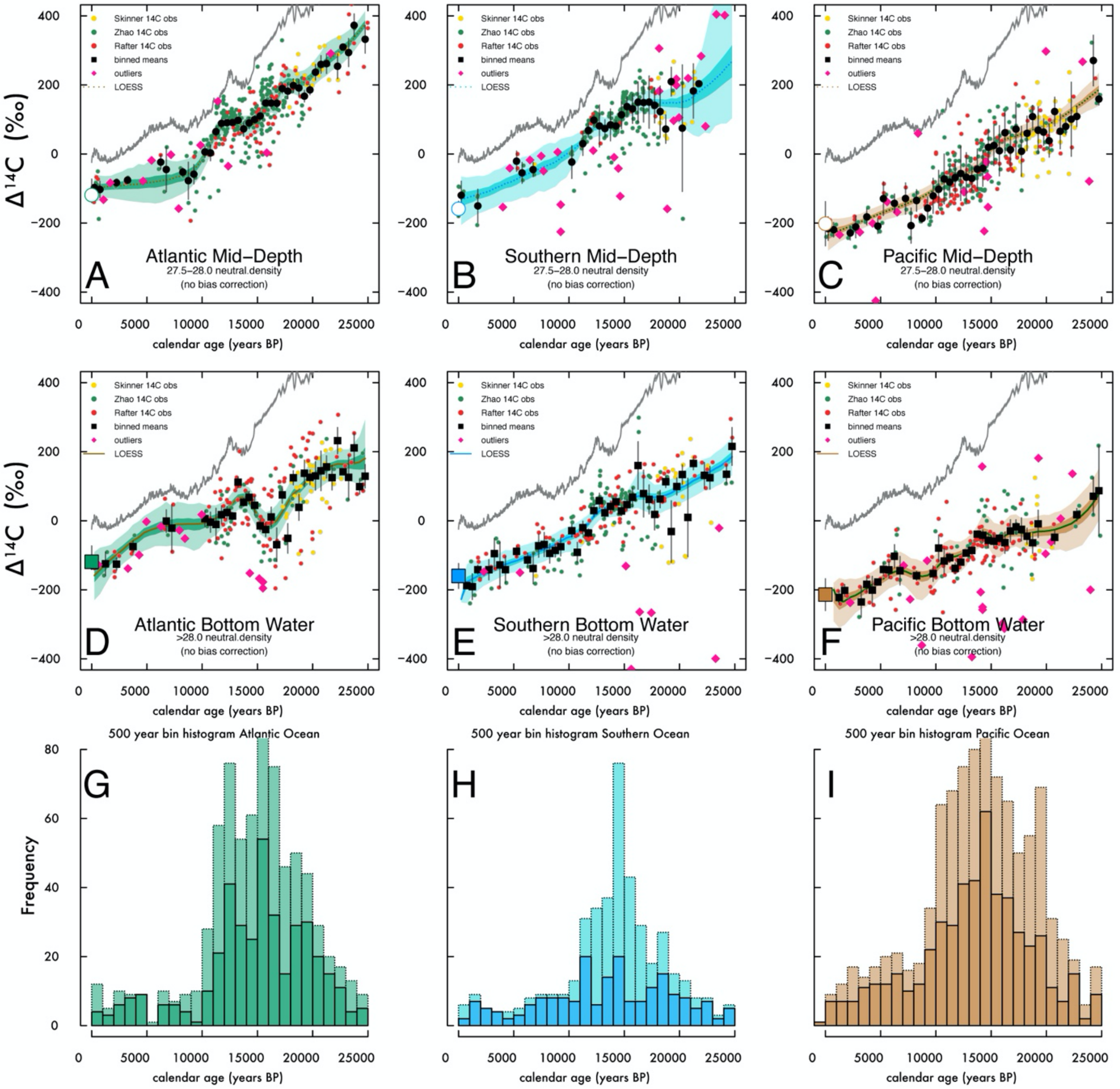

Figure S7: Mean seawater Δ^{14}C trends for Atlantic, Southern, and Pacific Oceans (left-to-right) without site bias correction. Here we show the LOESS trends of Δ^{14}C (in per mil) from Fig. 3 plus all the observations (grey circles), sorted by their published compilation (see legend). Black symbols and lines are binned mean values and 1-standard error, respectively. Pink diamonds are observations that are >3-standard deviation the binned mean or bins with only one observation (these outliers are not used in the LOESS estimate). Panels (A-C) are for mid-depth density surfaces and panels (D-F) are for bottom water density surfaces. Panels (G-I) are the histograms for the observations used to create the LOESS trends, dotted outline bars are mid-depth and solid outline bars are bottom-waters. There was no depth-bias correction applied to these observations (see Methods). LOESS error envelopes show 68% and 95% confidence interval from bootstrapping/Monte-Carlo simulation (see Methods).

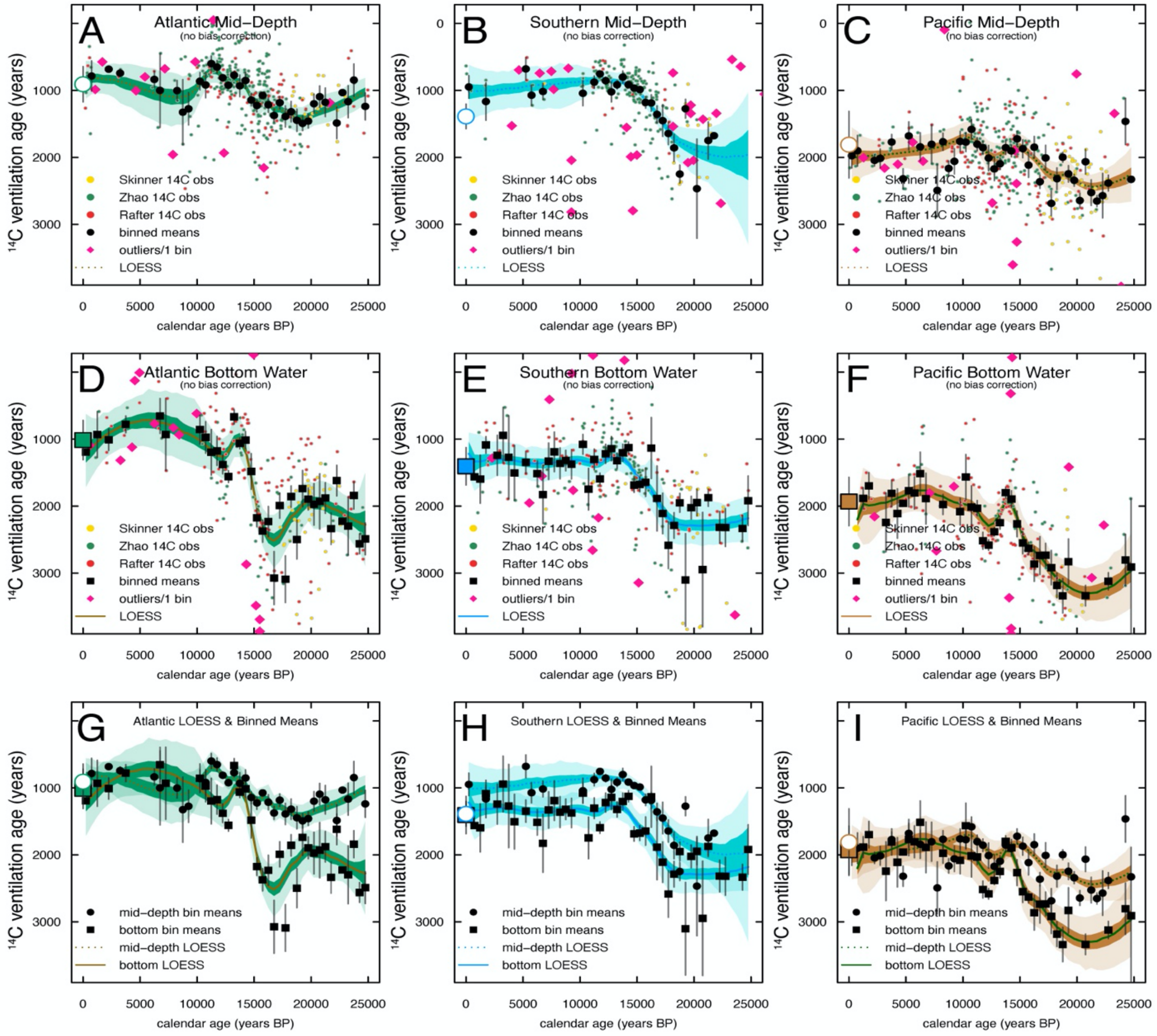

Figure S8. Trends and binned mean values for ^{14}C ventilation age in the Atlantic, Southern, and Pacific Oceans (without site bias correction). Here we show the LOESS trends of the ^{14}C ventilation age (years) from Fig. 3 plus all the observations (grey circles). Black symbols and lines show binned mean values used for calculating the LOESS trends and 1-standard error, respectively. Pink diamonds are observations that are >3-standard deviation the binned mean or bins with only one observation (these outliers are not used in the LOESS estimate). Panels (A-C) are for mid-depth density surfaces and panels (D-F) are for bottom water density surfaces. Panels (G-I) show all the data together and is included to illustrate the ^{14}C ventilation age differences between mid-depth and bottom waters, regardless of analysis. In all figures, dashed lines and circles are mid-depth and solid lines and squares are bottom waters. There was no depth-bias correction applied to these observations (see Methods). LOESS error envelopes show 68% and 95% confidence interval from bootstrapping/Monte-Carlo simulation (see Methods).

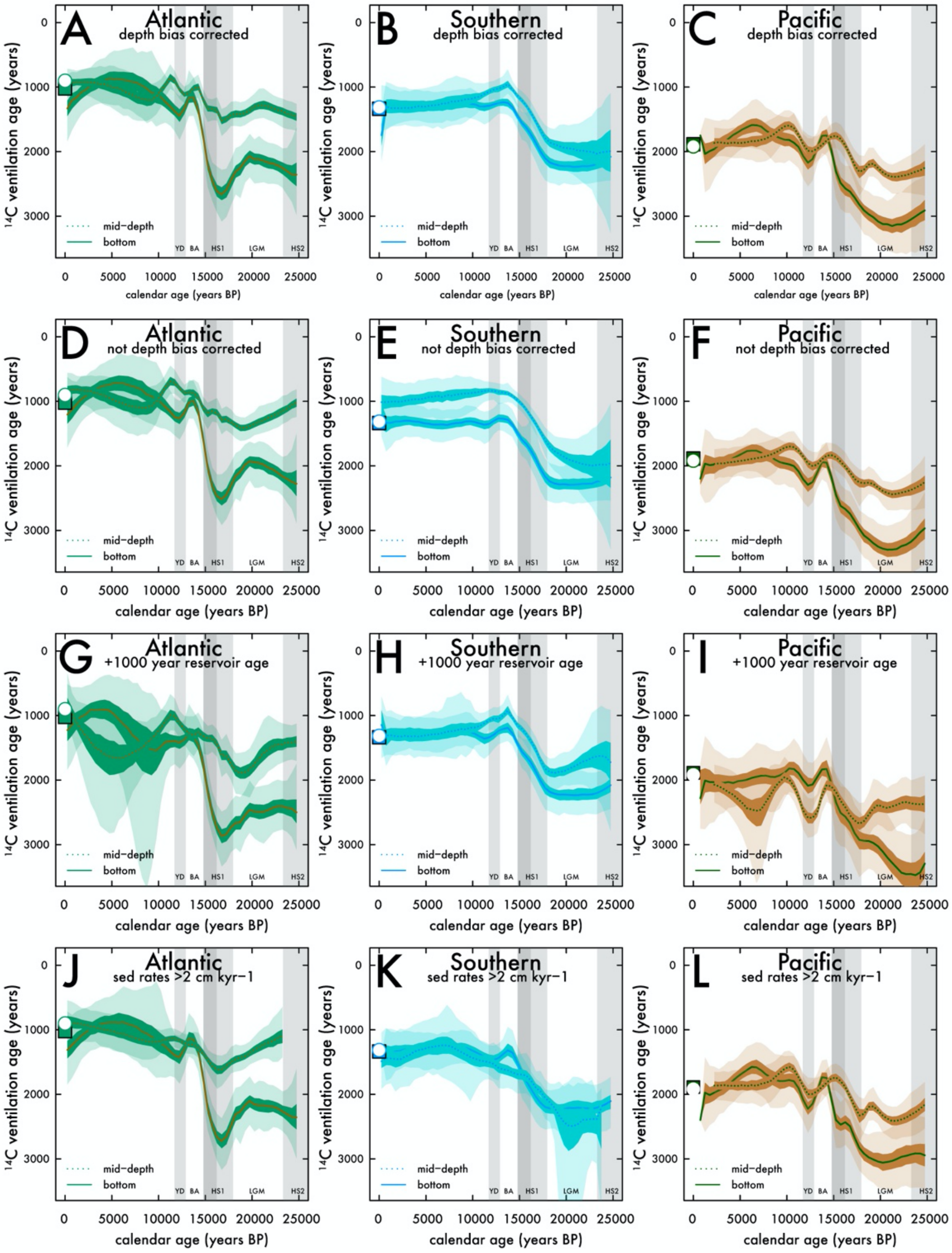

Figure S9. Sensitivity tests of basin average ^{14}C ventilation age trends.
(A-C) Trends for data with depth-bias correction and using γ^n separation of 27.5 and 28 kg m^{-3}, the same as in Fig. 3 and included here for comparison. **(D-F)** Same as (A), but without depth bias correction. **(G-I)** Same data as (A-C), but assuming a +1000-year additional reservoir age for sites using planktic foram-based age models, applied from 25-to-10-kyr BP. **(J-L)** Same data as (A-C), but without core sites with sedimentation rates <2 cm kyr^{-1}. Symbols are modern DIC ^{14}C age for mid-depth (circle) and bottom water (square).

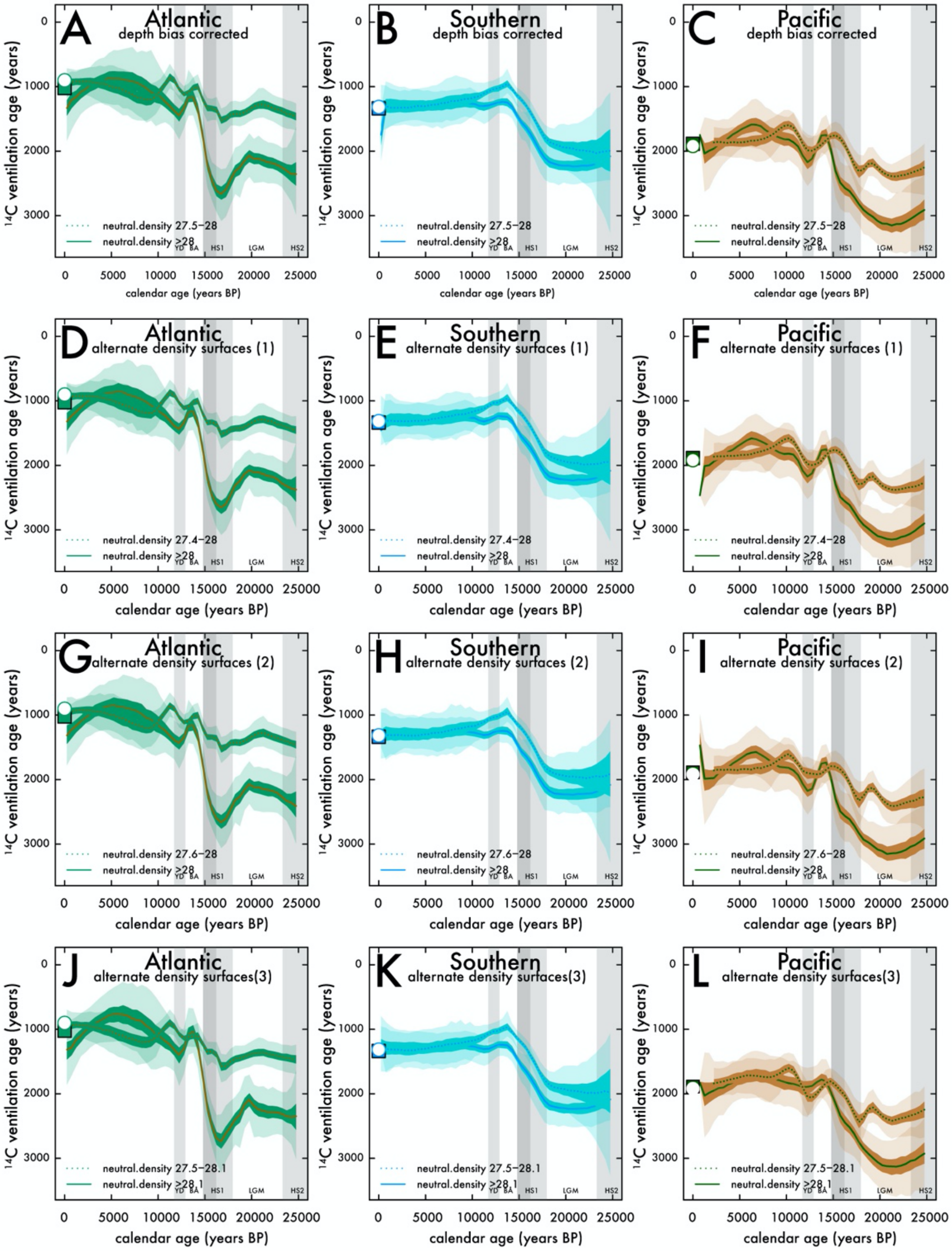

Figure S10: Sensitivity tests of basin average ^{14}C ventilation age trends for variable neutral density (γ^n) surfaces. (A-C) Trends for data with depth-bias correction and using γ^n separation of 27.5 and 28 kg m^{-3}, the same as in Fig. 3 and included here for comparison. **(D-F)** Same data as (A-C), but using γ^n separation of 27.4 and 28 kg m^{-3}. **(G-I)** Same data as (A-C), but using γ^n separation of 27.6 and 28 kg m^{-3}. **(J-L)** Same data as (A-C), but using γ^n separation of 27.5 and 28.1 kg m^{-3}. Symbols are modern DIC ^{14}C age for mid-depth (circle) and bottom water (square).

REFERENCES AND NOTES

1. M. P. Hain, D. M. Sigman, G. H. Haug, Distinct roles of the Southern Ocean and North Atlantic in the deglacial atmospheric radiocarbon decline. *Earth Planet. Sci. Lett.* **394**, 198–208 (2014).

2. A. W. Jacobel, R. F. Anderson, S. L. Jaccard, J. F. McManus, F. J. Pavia, G. Winckler, Deep Pacific storage of respired carbon during the last ice age: Perspectives from bottom water oxygen reconstructions. *Quat. Sci. Rev.* **230**, 106065 (2020).

3. S. L. Jaccard, E. D. Galbraith, A. Martínez-García, R. F. Anderson, Covariation of deep Southern Ocean oxygenation and atmospheric CO_2 through the last ice age. *Nature* **530**, 207–210 (2016).

4. B. A. A. Hoogakker, Z. Lu, N. Umling, L. Jones, X. Zhou, R. E. M. Rickaby, R. Thunell, O. Cartapanis, E. Galbraith, Glacial expansion of oxygen-depleted seawater in the eastern tropical Pacific. *Nature* **562**, 410–413 (2018).

5. L. C. Skinner, S. Fallon, C. Waelbroeck, E. Michel, S. Barker, Ventilation of the deep Southern ocean and deglacial CO_2 rise. *Science* **328**, 1147–1151 (2010).

6. M. Sarnthein, B. Schneider, P. M. Grootes, Peak glacial ^{14}C ventilation ages suggest major draw-down of carbon into the abyssal ocean. *Clim. Past.* **9**, 2595–2614 (2013).

7. A. Schmittner, E. D. Galbraith, Glacial greenhouse-gas fluctuations controlled by ocean circulation changes. *Nature* **456**, 373–376 (2008).

8. G. Gebbie, P. Huybers, Meridional circulation during the Last Glacial Maximum explored through a combination of South Atlantic $\delta^{18}O$ observations and a geostrophic inverse model. *Geochem. Geophys. Geosyst.* **7**, 1–5 (2006).

9. P. LeGrand, C. Wunsch, Constraints from paleotracer data on the North Atlantic circulation during the Last Glacial Maximum. *Paleoceanography* **10**, 1011–1045 (1995).

10. S. Khatiwala, A. Schmittner, J. Muglia, Air-sea disequilibrium enhances ocean carbon storage during glacial periods. *Sci. Adv.* **5**, eaaw4981 (2019).

11. E. D. Galbraith, L. C. Skinner, The biological pump during the Last Glacial Maximum. *Ann. Rev. Mar. Sci.* **12**, 559–586 (2020).

12. B. B. Stephens, R. F. Keeling, The influence of Antarctic sea ice on glacial-interglacial CO_2 variations. *Nature* **404**, 171–174 (2000).

13. W. S. Broecker, R. D. Gerard, M. Ewing, B. C. Heezen, "Geochemistry and physics of ocean circulation" (NYO-2921; CU-6-59-1808-Geol., Columbia Univ., Palisades, N.Y. Lamont Geological Observatory, United States, 1959).

14. D. C. Lund, F. J. Pavia, E. I. Seeley, S. E. McCart, P. A. Rafter, K. A. Farley, P. D. Asimow, R. F. Anderson, Hydrothermal scavenging of ^{230}Th on the Southern East Pacific Rise during the last deglaciation. *Earth Planet. Sci. Lett.* **510**, 64–72 (2019).

15. L. D. Stott, J. Southon, A. Timmermann, A. Koutavas, Radiocarbon age anomaly at intermediate water depth in the Pacific Ocean during the last deglaciation. *Paleoceanography* **24**, 1–10 (2009).

16. J. F. Adkins, E. A. Boyle, Changing atmospheric Δ^{14}C and the record of deepwater paleoventilation ages. *Geophys. Res. Lett.* **12**, 337–344 (1997).

17. S. K. V. Hines, J. R. Southon, J. F. Adkins, A high-resolution record of Southern Ocean intermediate water radiocarbon over the past 30,000 years. *Earth Planet. Sci. Lett.* **432**, 46–58 (2015).

18. G. Soulet, L. C. Skinner, S. R. Beaupré, V. Galy, A note on reporting of reservoir ^{14}C disequilibria and age offsets. *Radiocarbon* **58**, 205–211 (2016).

19. M. S. Cook, L. D. Keigwin, Radiocarbon profiles of the NW Pacific from the LGM and deglaciation: Evaluating ventilation metrics and the effect of uncertain surface reservoir ages. *Paleoceanography* **30**, 174–195 (2015).

20. P. J. Reimer, W. E. N. Austin, E. Bard, A. Bayliss, P. G. Blackwell, C. B. Ramsey, M. Butzin, H. Cheng, R. L. Edwards, M. Friedrich, P. M. Grootes, T. P. Guilderson, I. Hajdas, T. J. Heaton, A. G. Hogg, K. A. Hughen, B. Kromer, S. W. Manning, R. Muscheler, J. G. Palmer, C. Pearson, J. van der Plicht, R. W. Reimer, D. A. Richards, E. M. Scott, J. R. Southon, C. S. M. Turney, L. Wacker, F.

Adolphi, U. Büntgen, M. Capano, S. M. Fahrni, A. Fogtmann-Schulz, R. Friedrich, P. Köhler, S. Kudsk, F. Miyake, J. Olsen, F. Reinig, M. Sakamoto, A. Sookdeo, S. Talamo, The IntCal20 northern hemisphere radiocarbon age calibration curve (0–55 cal kBP). *Radiocarbon* **62**, 725–757 (2020).

21. N. Zhao, O. Marchal, L. Keigwin, D. Amrhein, G. Gebbie, A synthesis of deglacial deep-sea radiocarbon records and their (In)consistency with modern ocean ventilation. *Paleoceanogr. Paleoclimatol.* **33**, 128–151 (2018).

22. W. S. Broecker, The great ocean conveyor. *Oceanography* **4**, 11 (1991).

23. L. Talley, Closure of the global overturning circulation through the Indian, Pacific, and Southern Oceans: Schematics and transports. *Oceanography* **26**, 80–97 (2013).

24. V. Tamsitt, H. F. Drake, A. K. Morrison, L. D. Talley, C. O. Dufour, A. R. Gray, S. M. Griffies, M. R. Mazloff, J. L. Sarmiento, J. Wang, W. Weijer, Spiraling pathways of global deep waters to the surface of the Southern Ocean. *Nat. Commun.* **8**, 172 (2017).

25. J. L. Sarmiento, N. Gruber, M. A. Brzezinski, J. P. Dunne, High-latitude controls of thermocline nutrients and low latitude biological productivity. *Nature* **427**, 56–60 (2004).

26. K. Matsumoto, Radiocarbon-based circulation age of the world oceans. *J. Geophys. Res.* **112**, C09004 (2007).

27. J. W. B. Rae, W. Broecker, What fraction of the Pacific and Indian Oceans' deep water is formed in the Southern Ocean? *Biogeosciences* **15**, 3779–3794 (2018).

28. R. Lumpkin, K. Speer, Global ocean meridional overturning. *J. Phys. Oceanogr.* **37**, 2550–2562 (2007).

29. J. Marshall, K. Speer, Closure of the meridional overturning circulation through Southern Ocean upwelling. *Nat. Geosci.* **5**, 171–180 (2012).

30. A. Olsen, N. Lange, R. M. Key, T. Tanhua, M. Álvarez, S. Becker, H. C. Bittig, B. R. Carter, L. Cotrim da Cunha, R. A. Feely, S. van Heuven, M. Hoppema, M. Ishii, E. Jeansson, S. D. Jones, S.

Jutterström, M. K. Karlsen, A. Kozyr, S. K. Lauvset, C. Lo Monaco, A. Murata, F. F. Pérez, B. Pfeil, C. Schirnick, R. Steinfeldt, T. Suzuki, M. Telszewski, B. Tilbrook, A. Velo, R. Wanninkhof, GLODAPv2.2019—An update of GLODAPv2. *Earth Syst. Sci. Data* **11**, 1437–1461 (2019).

31. E. Bard, Geochemical and geophysical implications of the radiocarbon calibration. *Geochim. Cosmochim. Acta* **62**, 2025–2038 (1998).

32. E. D. Galbraith, E. Y. Kwon, D. Bianchi, M. P. Hain, J. L. Sarmiento, The impact of atmospheric $p\mathrm{CO_2}$ on carbon isotope ratios of the atmosphere and ocean. *Global Biogeochem. Cycles* **29**, 307–324 (2015).

33. L. C. Skinner, F. Primeau, E. Freeman, M. de la Fuente, P. A. Goodwin, J. Gottschalk, E. Huang, I. N. McCave, T. L. Noble, A. E. Scrivner, Radiocarbon constraints on the glacial ocean circulation and its impact on atmospheric CO_2. *Nat. Commun.* **8**, 16010 (2017).

34. D. M. Sigman, E. A. Boyle, Glacial/interglacial variations in atmospheric carbon dioxide. *Nature* **407**, 859–869 (2000).

35. L. D. Keigwin, Glacial-age hydrography of the far northwest pacific. *Paleoceanography* **13**, 323–339 (1998).

36. J. C. Herguera, T. Herbert, M. Kashgarian, C. Charles, Intermediate and deep water mass distribution in the Pacific during the Last Glacial Maximum inferred from oxygen and carbon stable isotopes. *Quat. Sci. Rev.* **29**, 1228–1245 (2010).

37. J. D. Carriquiry, A. Sanchez, G. Leduc, Southern Ocean influence on the eastern tropical North Pacific's intermediate-depth circulation during the Last Glacial Maximum. *Paleoceanography* **30**, 1132–1151 (2015).

38. L.-P. Nadeau, R. Ferrari, M. F. Jansen, Antarctic sea ice control on the depth of north atlantic deep water. *J. Climate* **32**, 2537–2551 (2019).

39. J. Du, B. A. Haley, A. C. Mix, Evolution of the global overturning circulation since the Last Glacial Maximum based on marine authigenic neodymium isotopes. *Quat. Sci. Rev.* **241**, 106396 (2020).

40. W. B. Curry, D. W. Oppo, Glacial water mass geometry and the distribution of $\delta^{13}C$ of ΣCO_2 in the western Atlantic Ocean. *Paleoceanography* **20**, PA1017 (2005).

41. D. C. Lund, A. C. Mix, J. Southon, Increased ventilation age of the deep northeast Pacific Ocean during the last deglaciation. *Nat. Geosci.* **4**, 771–774 (2011).

42. J. W. B. Rae, W. R. Gray, R. C. J. Wills, I. Eisenman, B. Fitzhugh, M. Fotheringham, E. F. M. Littley, P. A. Rafter, R. Rees-Owen, A. Ridgwell, B. Taylor, A. Burke, Overturning circulation, nutrient limitation, and warming in the Glacial North Pacific. *Sci. Adv.* **6**, eabd1654 (2020).

43. J. W. B. Rae, M. Sarnthein, G. L. Foster, A. Ridgwell, P. M. Grootes, T. Elliott, Deep water formation in the North Pacific and deglacial CO_2 rise. *Paleoceanography* **29**, 645–667 (2014).

44. Y. Okazaki, A. Timmermann, L. Menviel, N. Harada, A. Abe-Ouchi, M. O. Chikamoto, A. Mouchet, H. Asahi, Deepwater formation in the north pacific during the last glacial termination. *Science* **329**, 200–204 (2010).

45. A. Martinez-Garcia, D. M. Sigman, H. Ren, R. F. Anderson, M. Straub, D. A. Hodell, S. L. Jaccard, T. I. Eglinton, G. H. Haug, Iron fertilization of the Subantarctic ocean during the last ice age. *Science* **343**, 1347–50 (2014).

46. A. Burke, L. Robinson, The Southern Ocean's role in carbon exchange during the last deglaciation. *Science* **335**, 557–561 (2012).

47. J. W. B. Rae, A. Burke, L. F. Robinson, J. F. Adkins, T. Chen, C. Cole, R. Greenop, T. Li, E. F. M. Littley, D. C. Nita, J. A. Stewart, B. J. Taylor, CO_2 storage and release in the deep Southern Ocean on millennial to centennial timescales. *Nature* **562**, 569–573 (2018).

48. J. Yu, W. S. Broecker, H. Elderfield, Z. Jin, J. McManus, F. Zhang, Loss of carbon from the deep sea since the Last Glacial Maximum. *Science* **330**, 1084–1087 (2010).

49. L. F. Robinson, J. F. Adkins, L. D. Keigwin, J. Southon, D. P. Fernandez, S.-L. Wang, D. S. Scheirer, Radiocarbon variability in the western north atlantic during the last deglaciation. *Science* **310**, 1469–1473 (2005).

50. J. F. McManus, R. Francois, J.-M. Gherardi, L. D. Keigwin, S. Brown-Leger, Collapse and rapid resumption of Atlantic meridional circulation linked to deglacial climate changes. *Nature* **428**, 834–837 (2004).

51. B. A. A. Hoogakker, H. Elderfield, G. Schmiedl, I. N. McCave, R. E. M. Rickaby, Glacial–Interglacial changes in bottom-water oxygen content on the Portuguese margin. *Nat. Geosci.* **8**, 40–43 (2015).

52. R. F. Anderson, S. Ali, L. I. Bradtmiller, S. H. Nielsen, M. Q. Fleisher, B. E. Anderson, L. H. Burckle, Wind-driven upwelling in the Southern Ocean and the deglacial rise in atmospheric CO_2. *Science* **323**, 1443–8 (2009).

53. T. Li, L. F. Robinson, T. Chen, X. T. Wang, A. Burke, J. W. B. Rae, A. Pegrum-Haram, T. D. J. Knowles, G. Li, J. Chen, H. C. Ng, M. Prokopenko, G. H. Rowland, A. Samperiz, J. A. Stewart, J. Southon, P. T. Spooner, Rapid shifts in circulation and biogeochemistry of the Southern Ocean during deglacial carbon cycle events. *Sci. Adv.* **6**, eabb3807 (2020).

54. L. C. Skinner, E. Freeman, D. Hodell, C. Waelbroeck, N. Vazquez Riveiros, A. E. Scrivner, Atlantic ocean ventilation changes across the last deglaciation and their carbon cycle implications. *Paleoceanogr. Paleoclimatol.* **36**, e2020PA004074 (2021).

55. L. D. Keigwin, Radiocarbon and stable isotope constraints on Last Glacial Maximum and Younger Dryas ventilation in the western North Atlantic. *Paleoceanography* **19**, 1–15 (2004).

56. D. C. Lund, P. D. Asimow, Does sea level influence mid-ocean ridge magmatism on Milankovitch timescales? *Geochem. Geophys. Geosystems* **12**, 1–26 (2011).

57. P. A. Rafter, J. C. Herguera, J. R. Southon, Extreme lowering of deglacial seawater radiocarbon recorded by both epifaunal and infaunal benthic foraminifera in a wood-dated sediment core. *Clim. Past* **14**, 1977–1989 (2018).

58. P. A. Rafter, J. D. Carriquiry, J. Herguera, M. P. Hain, E. A. Solomon, J. R. Southon, Anomalous >2000 year old surface ocean radiocarbon age as evidence for deglacial geologic carbon release. *Geophys. Res. Lett.* **46**, 2019GL085102 (2019).

59. T. Chen, L. F. Robinson, A. Burke, L. Claxton, M. P. Hain, T. Li, J. W. B. Rae, J. Stewart, T. D. J. Knowles, D. J. Fornari, K. S. Harpp, Persistently well-ventilated intermediate-depth ocean through the last deglaciation. *Nat. Geosci.* **13**, 733–738 (2020).

60. N. Bharti, R. Bhushan, L. Skinner, M. Muruganantham, P. S. Jena, A. Dabhi, A. Shivam, Evidence of poorly ventilated deep Central Indian Ocean during the last glaciation. *Earth Planet. Sci. Lett.* **582**, 117438 (2022).

61. B. Bereiter, S. Eggleston, J. Schmitt, C. Nehrbass-Ahles, T. F. Stocker, H. Fischer, S. Kipfstuhl, J. Chappellaz, Revision of the EPICA Dome C CO_2 record from 800 to 600 kyr before present. *Geophys. Res. Lett.* **42**, 542–549 (2015).

62. W. Libby, *Radiocarbon Dating* (University of Chicago Press, ed. 2, 1955).

63. H. Godwin, Half-life of radiocarbon. *Nature* **195**, 984 (1962).

64. J. F. Adkins, H. Cheng, E. A. Boyle, E. R. M. Druffel, L. R. Edwards, Deep-sea coral evidence for rapid change in ventilation of the deep North Atlantic 15,400 years ago. *Science* **280**, 725–728 (1998).

65. T. J. Heaton, P. Köhler, M. Butzin, E. Bard, R. W. Reimer, W. E. N. Austin, C. Bronk Ramsey, P. M. Grootes, K. A. Hughen, B. Kromer, P. J. Reimer, J. Adkins, A. Burke, M. S. Cook, J. Olsen, L. C. Skinner, Marine20—The marine radiocarbon age calibration curve (0–55,000 cal BP). *Radiocarbon* **62**, 779–820 (2020).

66. M. Butzin, P. Köhler, G. Lohmann, Marine radiocarbon reservoir age simulations for the past 50,000 years. *Geophys. Res. Lett.* **44**, 8473–8480 (2017).

67. M. Blaauw, J. A. Christen, Flexible paleoclimate age-depth models using an autoregressive gamma process. *Bayesian Anal.* **6**, 457–474 (2011).

68. W. Broecker, E. Clark, Radiocarbon-age differences among coexisting planktic foraminifera shells: The barker effect. *Paleoceanography* **26**, PA2222 (2011).

69. S. Barker, W. Broecker, E. Clark, I. Hajdas, Radiocarbon age offsets of foraminifera resulting from differential dissolution and fragmentation within the sedimentary bioturbated zone: RADIOCARBON AGE OFFSETS. *Paleoceanography* **22**, PA2205 (2007).

70. W. S. Broecker, M. Klas, N. Ragano-Beavan, G. Mathieu, A. Mix, Accelerator mass spectrometry radiocarbon measurements on marine carbonate samples from deep sea cores and sediment traps. *Radiocarbon* **30**, 261–263 (1988).

71. W. S. Broecker, M. Andree, G. Bonani, W. Wolfli, H. Oeschger, M. Klas, A. Mix, W. Curry, Preliminary estimates for the radiocarbon age of deep water in the glacial ocean. *Paleoceanography* **3**, 659–669 (1988).

72. A. L. Magana, J. R. Southon, J. P. Kennett, E. B. Roark, M. Sarnthein, L. D. Stott, Resolving the cause of large differences between deglacial benthic foraminifera radiocarbon measurements in Santa Barbara Basin. *Paleoceanography* **25**, 1–8 (2010).

73. M. M. Ezat, T. L. Rasmussen, D. J. R. Thornalley, J. Olsen, L. C. Skinner, B. Hönisch, J. Groeneveld, Ventilation history of Nordic Seas overflows during the last (de)glacial period revealed by species-specific benthic foraminiferal ^{14}C dates. *Paleoceanography* **32**, 172–181 (2017).

74. G. M. Santos, R. B. Moore, J. R. Southon, S. Griffin, E. Hinger, D. Zhang, AMS ^{14}C sample preparation at the KCCAMS/UCI facility: Status report and performance of small samples. *Radiocarbon* **49**, 255–269 (2007).

75. J. Southon, G. Santos, K. Druffel-Rodriguez, E. Druffel, S. Trumbore, X. Xu, S. Griffin, S. Ali, M. Mazon, The Keck carbon cycle AMS laboratory, University of California, Irvine: Initial operation and a background surprise. *Radiocarbon* **46**, 41–49 (2004).

76. E. R. Druffel, Bomb radiocarbon in the Pacific: Annual and seasonal timescale variations. *J. Mar. Res.* **45**, 667–698 (1987).

77. R. M. Key, A. Kozyr, C. L. Sabine, K. Lee, R. Wanninkhof, J. L. Bullister, R. A. Feely, F. J. Millero, C. Mordy, T. H. Peng, A global ocean carbon climatology: Results from global data analysis project (GLODAP). *Global Biogeochem. Cycles* **18**, 1–23 (2004).

78. A. Burke, A. L. Stewart, J. F. Adkins, R. Ferrari, M. F. Jansen, A. F. Thompson, The glacial mid-depth radiocarbon bulge and its implications for the overturning circulation. *Paleoceanography* **30**, 1021–1039 (2015).

79. T. DeVries, M. Holzer, Radiocarbon and helium isotope constraints on deep ocean ventilation and mantle-^{3}He sources. *J. Geophys. Res. Oceans.* **124**, 3036–3057 (2019).

80. M. S. Cook, L. D. Keigwin, D. Birgel, K.-U. Hinrichs, Repeated pulses of vertical methane flux recorded in glacial sediments from the southeast Bering Sea. *Paleoceanography* **26**, 1–17 (2011).

81. L. D. Keigwin, T. P. Guilderson, Bioturbation artifacts in zero-age sediments. *Paleoceanography* **24**, 1–6 (2009).

82. J. Wycech, D. C. Kelly, S. Marcott, Effects of seafloor diagenesis on planktic foraminiferal radiocarbon ages. *Geology* **44**, 551–554 (2016).

83. M. Davies-Walczak, A. C. Mix, J. S. Stoner, J. R. Southon, M. Cheseby, C. Xuan, Late glacial to holocene radiocarbon constraints on North Pacific Intermediate Water ventilation and deglacial atmospheric CO_2 sources. *Earth Planet. Sci. Lett.* **397**, 57–66 (2014).

84. V. Tamsitt, R. P. Abernathey, M. R. Mazloff, J. Wang, L. D. Talley, Transformation of deep water masses along lagrangian upwelling pathways in the Southern Ocean. *J. Geophys. Res. Oceans.* **123**, 1994–2017 (2018).

85. B. Efron, Computers and the theory of statistics: Thinking the unthinkable. *SIAM Rev.* **21**, 460–480 (1979).

86. Y. Dai, J. Yu, P. Rafter, Deglacial ventilation changes in the deep Southwest Pacific. *Paleoceanogr. Paleoclimatol.* **36**, e2020PA004172 (2021).

87. R. Schlitzer, Ocean data view (2002); https://odv.awi.de.

88. P. A. Rafter, Color palettes for ocean data view (2019); www.prafter.com/color.

89. R. A. Locarnini, A. V. Mishonov, O. K. Baranova, T. P. Boyer, M. M. Zweng, H. E. Garcia, J. R. Reagan, K. W. Seidov, K. W. Weathers, C. R. Paver, I. V. Smolyar, "World Ocean Atlas 2018, Volume 1: Temperature," *NOAA Atlas NESDIS 81* (Silver Spring, MD, 2019), p. 52.
